# Pharmacokinetics and Pharmacodynamics of Intranasal Solid Lipid Nanoparticles and Nanostructured Lipid Carriers for Nose-to-Brain Delivery

**DOI:** 10.3390/pharmaceutics14030572

**Published:** 2022-03-05

**Authors:** Thi-Thao-Linh Nguyen, Han-Joo Maeng

**Affiliations:** College of Pharmacy, Gachon University, 191 Hambakmoe-ro, Yeonsu-gu, Incheon 21936, Korea; linh.nguyen@gachon.ac.kr

**Keywords:** nose-to-brain, drug delivery, SLNs, NLCs, central nervous system, blood-brain barrier, brain targeting, pharmacokinetics, pharmacodynamics

## Abstract

Nose-to-brain drug delivery has been of great interest for the treatment of many central nervous system (CNS) diseases and psychiatric disorders over past decades. Several nasally administered formulations have been developed to circumvent the blood-brain barrier and directly deliver drugs to the CNS through the olfactory and trigeminal pathways. However, the nasal mucosa’s drug absorption is insufficient and the volume of the nasal cavity is small, which, in combination, make nose-to-brain drug delivery challenging. These problems could be minimized using formulations based on solid lipid nanoparticles (SLNs) or nanostructured lipid carriers (NLCs), which are effective nose-to-brain drug delivery systems that improve drug bioavailability by increasing drug solubility and permeation, extending drug action, and reducing enzymatic degradation. Various research groups have reported in vivo pharmacokinetics and pharmacodynamics of SLNs and NLCs nose-to-brain delivery systems. This review was undertaken to provide an overview of these studies and highlight research performed on SLN and NLC-based formulations aimed at improving the treatment of CNS diseases such neurodegenerative diseases, epilepsy, and schizophrenia. We discuss the efficacies and brain targeting efficiencies of these formulations based on considerations of their pharmacokinetic parameters and toxicities, point out some gaps in current knowledge, and propose future developmental targets.

## 1. Introduction

Drug development for central nervous system (CNS) diseases and psychiatric disorders is challenging due to the side effects of drugs, the complexity of the brain, and notably, the lack of efficient strategies to deliver drugs across the blood-brain barrier (BBB) [1,2]. The BBB is composed of tightly connected endothelial capillary cells and plays a critical role in protecting the CNS from pathogens and solutes in blood [3]. Solute molecules can cross the BBB via different mechanisms. Several lipid-soluble molecules can enter the brain by passive diffusion. In this mechanism, the molecule lipophilicity generally defines the penetration rate and extent into the brain. However, many of these molecules are usually pumped back to the circulatory system by some efflux pumps expressed in the BBB. Small polar molecules, such as amino acids, glucose, nucleosides, and organic anions and cations, are transported by carrier-mediated transport. Another mechanism is receptor-mediated transcytosis, which transports large molecules, such as iron, insulin, and leptin [4]. Similar to Lipinski’s rule of five, the permeation of a molecule across the BBB depends on its molecular weight, lipophilicity, H bond donors and acceptors, charge, and polar surface area [5,6]. Thus, only a small number of hydrophobic and low molecular weight molecules can cross the BBB, whereas others are restricted by the barrier characteristics of the BBB, which makes it difficult to develop drugs that target the brain [7].

During recent years, intranasal (IN) administration, a non-invasive drug delivery approach for local or systemic effects, has been used to provide direct nose-to-brain transport. Due to the presence of direct anatomical connection between the CNS and the nasal cavity, IN administration can provide the access of drugs to the CNS [8,9,10,11]. As compared to parenteral administration, IN delivery can overcome the BBB, provide faster brain delivery, and enhance drug targeting and drug bioavailability. Furthermore, IN administration avoids gastrointestinal and hepatic metabolisms, decreases drug accumulation in non-targeted organs, and reduces systemic side effects [12,13,14]. Therefore, nose-to-brain delivery is an effective approach to manage CNS diseases and psychiatric disorders.

Despite these advantages, IN administration for nose-to-brain transport has several limitations that must be overcome during the development of novel formulations. First, the administration volume for each nostril is limited (<200 µL) in humans, making this route challenging for drugs requiring high doses. Second, administered samples are easily lost due to mucociliary clearance, a primary defense mechanism that prevents pathogens, toxins, and particles from entering the body [15]. Thus, formulations may have short residence times (15–30 min) in the nasal cavity, which confines the drug absorption. Third, enzymes in the nasal cavity can metabolize a number of drugs, and thus, these drugs need to be protected from enzymatic degradation [16,17]. Forth, IN formulations must not cause irritation in the nasal cavity, i.e., these formulations need to have pH values and viscosities compatible with nasal mucosa and their components do not induce destruction or inflammation of the nasal epithelium [18]. In recent years, many formulation approaches including liposomes, emulsions, polymeric nanoparticles, inorganic nanoparticles, solid lipid nanoparticles (SLNs), and nanostructured lipid carriers (NLCs) have been developed to address these issues [19,20,21,22,23,24,25]. In particular, formulations based on SLNs and NLCs provide effective nose-to-brain transport to improve drug bioavailability by increasing drug solubility, drug permeation, and stability, extending the drug action, and reducing enzymatic degradation. SLNs and NLCs can also be loaded into hydrogel systems [26,27] or coated with mucoadhesive polymers to improve these effects [28,29].

Many research groups have successfully developed formulations based on SLNs and NLCs for nose-to-brain delivery with in vivo pharmacokinetic (PK) and pharmacodynamic (PD) studies. However, many reports did not calculate all PK parameters for the brain targeting and considerably evaluated them. Several reviews on SLNs and NLCs intranasal formulations have been published [30,31,32,33], which summarized results from some selected studies to highlight the efficacy of these formulations. However, none are systematic reviews to address the efficacy of nose-to-brain formulations based on in vivo PK and PD data. Therefore, in this review, we aimed to summarize these preclinical studies with an emphasis on the evaluation of brain targeting efficacy. We conducted a systematic review on in vivo nose-to-brain delivery of SLN and NLC-based formulations. We collected reported data or re-calculated data derived from PK studies to evaluate the brain targeting efficacies of formulations. By analysis of PK parameters, this review provides an insight into the brain targeting efficacies of SLN and NLC-based formulations and identifies their limitations. In addition, we present our perspectives for future development of these formulations.

## 2. Nose-to-Brain Delivery Pathways and Feasibilities of SLNs and NLCs for Nose-to-Brain Drug Delivery

### 2.1. Nose-to-Brain Delivery Pathways

**Figure 1:**
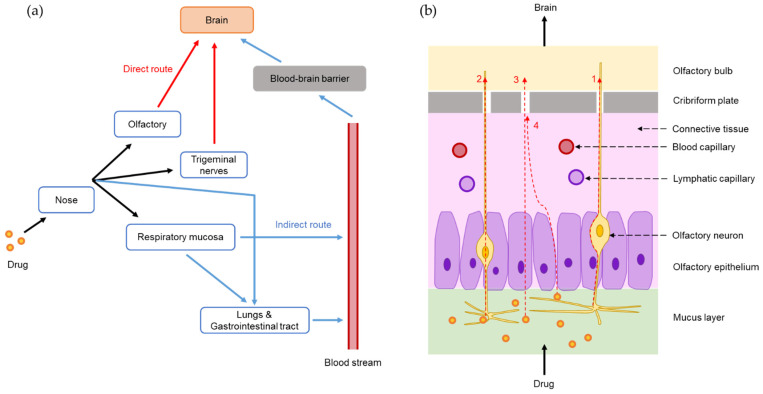
Pathways for nose-to-brain delivery of intranasal formulation: (**a**) Drug transports from the nose to the brain by direct route (olfactory and trigeminal pathways) and indirect (entering systemic circulation and crossing the blood-brain barrier). (**b**) Olfactory pathway for nose-to-brain delivery of drugs: (1) extraneuronal route, (2) intraneuronal route, (3) through supporting cells, and (4) along supporting cells.

Nose-to-brain delivery pathways have been extensively investigated and reported in many studies. As shown in Figure 1a, following the IN administration of a drug formulation, drug molecules or drug-loaded particles transport directly from the nose to the brain via olfactory and trigeminal nerves and indirectly into the systemic circulation before crossing the BBB to reach the brain [34,35,36].

When a drug enters the nasal cavity, it is distributed to the olfactory and respiratory regions. The olfactory region has olfactory nerves that begin at the olfactory epithelia and end at the olfactory bulb [37]. Drugs in the olfactory region can be transported to the brain by four different routes: (1) an extraneuronal route along olfactory neurons, (2) an intraneuronal route by olfactory neuron endocytosis, (3) through supporting cells by endocytosis, and (4) through the intercellular space by passing tight junctions (Figure 1b) [38,39]. The extraneuronal route (route 1) is the major direct pathway for drug transport from the nose to the brain, which may take up to 30 min. The intraneuronal route (route 2) involves drug endocytosis by olfactory neurons and release in the olfactory bulb before distributing to different brain regions, and this process may take several hours or days [7]. The drug transport through or along supporting cells (routes 3 and 4) is less important [39]. The olfactory pathway has been indicated in some studies. For example, fluorescence response to IN administered Cyanine7 NHS ester-loaded SLNs (Cys7-SLNs) was clearly observed in olfactory bulb, cerebellum, and striatum [12]. Furthermore, a proportion of drugs entering the respiratory region can be transported directly to the brain through the trigeminal nerve pathway via extraneuronal or intraneuronal routes although this pathway is less important than the olfactory pathway [7].

Drugs in the respiratory region are also absorbed into the bloodstream by respiratory epithelia. However, this process is only suitable for lipophilic drugs with low molecular weights and high BBB permeability. Drugs not absorbed in the nasal cavity can reach the lungs and gastrointestinal tract and subsequently be absorbed into the systemic circulation [7,40]. The drug may cross the BBB from the blood to reach the brain. However, this indirect pathway is less remarkable since the BBB prevents most drugs from reaching the brain, which is similar to any drug administered systemically [38].

### 2.2. Feasibilities of SLNs and NLCs for Nose-to-Brain Drug Delivery

SLNs and NLCs have been continuously developed over the last two decades. Both are colloidal nanoparticles with solid lipid matrices, which can be produced from solid lipids (SLNs) or mixtures of solid and liquid lipids (NLCs) [41,42,43]. SLNs and NLCs are alternatives to micelles, emulsions, liposomes, and polymeric nanoparticles in drug delivery. They have some distinct advantages that facilitate their wider applications for oral, parenteral, intranasal, ocular, transdermal, and pulmonary drug delivery [44,45,46]. The components of SLNs and NLCs are physiologically biocompatible and biodegradable lipids and other excipients that are generally recognized as safe (GRAS), making them safe nano-drug delivery systems [47,48]. SLNs and NLCs are more stable than micelles, emulsions, or liposomes because their solid matrices can protect incorporated drugs more efficiently [49]. Furthermore, SLNs and NLCs can encapsulate hydrophilic or hydrophobic drugs with higher entrapment efficiencies than liposomes [50,51]. Their lipid components can be modified to alter drug release [52,53]. NLCs have higher drug loadings and better drug stabilities during storage than SLNs because of their imperfect or amorphous structures, which provide more space to accommodate drugs [54,55,56].

SLNs and NLCs have several distinct advantages for nose-to-brain delivery. First, they can improve drug solubility and permeability, and the partition of nanoparticles into the lipid bilayer of the nasal epithelial cell membrane is greater than that of free drugs due to the lipophilic natures of SLNs and NLCs [57]. The nanosized particles with sufficient lipophilicity can easily squeeze through intercellular spaces between olfactory cells [58]. In addition, the use of surfactants (e.g., Tween 80, Tween 20, and sodium lauryl sulfate) can open tight junctions between epithelial cells and enhance drug permeability [59]. Second, incorporating drugs into SLNs and NLCs increases drug retention in the nasal cavity [60], and this retention can be improved by loading SLNs and NLCs into gel or coating them with suitable materials. Poloxamer 407, Poloxamer 188, methylcellulose, and hydroxypropyl methylcellulose (HPMC) are typically used for gel preparation. They also augment the nasal absorption of drugs [61,62]. Poloxamer can reduce mucus viscosity and elasticity, and, thereby, increase the transcellular transport of SLNs and NLCs [61]. Some mucoadhesive polymers, such as chitosan (CS) [63], trimethylchitosan [64], and glycol chitosan [29], can prolong the residence times of SLNs and NLCs in the nasal cavity. It was demonstrated in one study that a coating layer of polyethylene glycol 25 stearate inhibited P-glycoprotein efflux at cerebrovascular endothelial cell membranes, and thereby increased brain drug concentration [65]. Third, SLNs and NLCs can reduce the enzymatic degradation of drugs within nasal mucus better than solution-based dosage forms (e.g., solution, suspension, and gel) [58]. Finally, the safety of SLNs and NLCs well supports their feasibilities for nose-to-brain delivery [57].

## 3. In Vivo Evaluation of Intranasal Formulations for Nose-to-Brain Delivery

### 3.1. PK and Biodistribution Studies

Several different experimental designs have been used for PK studies to evaluate the IN administration of SLNs and NLCs for brain targeting. Generally, formulations (dispersions or gels) of SLNs or NLCs (administered IN) have been compared with free drug solutions or suspensions (IN), free drug solutions or suspensions (IV), and SLNs or NLCs (IV) in PK studies [65,66]. In some cases, oral administrations of free drug solutions or suspensions [67,68] or a marketed product [69] have also been used. However, the appropriate approach is to compare SLNs or NLCs (IN) with free drug solution or suspension (IN and IV) [15]. In PK and biodistribution studies, drug concentrations in blood and brain are determined at different time points, and areas under the curve for blood (AUC_blood_) and brain (AUC_brain_) are calculated for each formulation. In addition, maximum drug concentrations (C_max_) and times taken to reach these concentrations (T_max_) are also determined from PK profiles.

Different parameters can be compared among formulations to evaluate the brain targeting of an SLN and NLC-based formulation, such as AUC_brain_, C_max,brain_, T_max,brain_, drug targeting efficiency (DTE), drug transport percentage (DTP) [65], brain: blood ratios at each time point [70], and drug concentration in the brain at the final time point [71]. DTE and DTP are critical parameters of the brain targeting efficacies of nose-to-brain delivery systems. DTE% is a measure of drug accumulation in the brain following IN administration relative to IV administration, and is defined by:

(1) $$\mathrm{DTE}\%=\frac{\left({\mathrm{AUC}}_{\mathrm{brain},\mathrm{IN}}\right)/\left({\mathrm{AUC}}_{\mathrm{blood},\mathrm{IN}}\right)}{\left({\mathrm{AUC}}_{\mathrm{brain},\mathrm{IV}}\right)/\left({\mathrm{AUC}}_{\mathrm{blood},\mathrm{IV}}\right)}\times 100$$

where AUC is determined over the study duration (AUC_0–t_). DTE% values can range from 0 to ∞. A DTE% value of > or <100 suggests efficient or ineffective brain targeting, respectively. Log_10_ (DTE%) values are also used [15].

DTP% is the percentage of a drug that enters the brain via direct routes (through the olfactory and trigeminal pathways), and is defined by:

(2) $$\mathrm{DTP}\%=\frac{{\mathrm{AUC}}_{\mathrm{brain},\mathrm{IN}}-\left(\frac{{\mathrm{AUC}}_{\mathrm{brain},\mathrm{IV}}}{{\mathrm{AUC}}_{\mathrm{blood},\mathrm{IV}}}\times {\mathrm{AUC}}_{\mathrm{blood},\mathrm{IN}}\right)}{{\mathrm{AUC}}_{\mathrm{brain},\mathrm{IN}}}\times 100$$

The subtraction indicates the amount of drug entering the brain via the indirect route (from blood to the brain through the BBB). Equation (2) can be re-arranged and expressed as:

(3) $$\mathrm{DTP}\%=\left(1-\frac{100}{\mathrm{DTE}\%}\right)\times 100$$

DTP% values can range theoretically from −∞ to 100. When no drug is transported via direct routes, DTP% = 0. However, in some cases, DTP% values are <0 because DTE% values are <100. Positive DTP% values indicate drug delivery via direct routes contributes significantly to overall brain delivery. Drugs that do not easily cross the BBB have a high DTP% value, and a DTP% value of 100 indicates a drug cannot cross the BBB (AUC_brain,IV_ = 0) [15].

DTE% and DTP% values of drug-loaded SLNs and NLCs (IN) can be compared with those of the free drug solutions or suspensions (IN) using relative DTE% (logRDTE%) and relative DTP% (logRDTP%) as follows:

(4) $$\mathrm{LogRDTE}\%=\log_{10}\left(\frac{{\mathrm{DTE}\%}_{\mathrm{IN}\;\mathrm{SLNs}\;\mathrm{or}\;\mathrm{NLCs}}}{{\mathrm{DTE}\%}_{\mathrm{IN}\;\mathrm{free}\;\mathrm{drug}}}\times 100\right)$$

(5) $$\mathrm{LogRDTP}\%=\log_{10}\left(\frac{{\mathrm{DTP}\%}_{\mathrm{IN}\;\mathrm{SLNs}\;\mathrm{or}\;\mathrm{NLCs}}}{{\mathrm{DTP}\%}_{\mathrm{IN}\;\mathrm{free}\;\mathrm{drug}}}\times 100\right)$$

LogRDTE% and logRDTP% values of >2 indicate SLNs and NLCs (IN) have better brain targeting efficiencies than free drugs (IN) [15].

DTE% and DTP% are usually calculated using the AUC values of free drugs (IN), but in some cases, the AUC values of SLNs or NLCs (IN) have been used to calculate DTE% and DTP% values. To eliminate this inconsistency, we recalculated DTE% and DTP% values mentioned in these studies.

DTE% and DTP% are effective parameters for evaluating brain targeting by IN formulations, but if AUC_blood,IN_ is very low, DTE% and DTP% can be high despite a low AUC_brain,IN_. Therefore, other parameters are also used. B_IN/IV_ is the ratio of drug accumulation in the brain following IN administration over that following IV administration, and is calculated using:

(6) $${B\%}_{\mathrm{IN}/\mathrm{IV}}=\frac{{\mathrm{AUC}}_{\mathrm{brain},\mathrm{IN}}}{{\mathrm{AUC}}_{\mathrm{brain},\mathrm{IV}}}\times 100$$

A B%_IN/IV_ value of >100 indicates that IN administration results in greater accumulation of drug in brain than IV administration.

RB% is the ratio of drug accumulation in the brain for SLNs and NLCs (IN) versus that of the free drug (IN), and is also used to evaluate the effectiveness of brain targeting of SLNs and NLCs.

(7) $$\mathrm{RB}\%=\frac{{\mathrm{AUC}}_{\mathrm{brain},\mathrm{SLNs}\;\mathrm{and}\;\mathrm{NLCs}\;\left(\mathrm{IN}\right)}}{{\mathrm{AUC}}_{\mathrm{brain},\mathrm{free}\;\mathrm{drug}\;\left(\mathrm{IN}\right)}}\times 100$$

An RB% value of >100 indicates brain drug accumulation is higher for SLNs and NLCs (IN) than for the free drug (IN) [15].

Drug accumulation in the brain can be observed using gamma scintigraphy images after labeling the drug, SLNs, or NLCs with ^99m^Tc [66,72]. This technique is also used in PK studies to quantify drug accumulation in blood and the brain [73,74,75]. Fluorescence imaging is also used for the DiR DiIC18-labeled CS-NLCs [76]. SLNs and NLCs can be labeled with coumarin 6, or rhodamine-123 and visualized in brain tissue by confocal laser scanning microscopy [77,78].

### 3.2. Pharmacodynamic Studies

PD studies are performed to evaluate the efficacies of SLNs and NLCs (IN) in animals. Depending on drug and disease studies, appropriate animal models are selected to compare SLN or NLC candidates with free drugs or commercial products (administered IN, IV, or orally). For example, Plasmodium berghei ANKA-injected mice are used to evaluate the anti-cerebral malarial effects of artesunate [57], artemether, and lumefantrine [64]. In epilepsy, the maximal electroshock seizure model [79,80,81] or pentylenetetrazole-induced epilepsy rat model [82,83,84] are used. Treatment effects on Alzheimer’s disease and dementia are assessed using animals with scopolamine-induced amnesia [85,86,87,88,89] or streptozocin-induced Alzheimer’s disease [90]. Tests used for assessing depression include the tail suspension, the forced swimming [91], the locomotor activity [92], and the marble-burying tests [93]. Models used for Parkinson’s disease include 1-methyl-4-phenyl-1,2,3,6-tetrahydropyridine-induced Parkinson’s disease [94], 6-hydroxydopamine partially lesioned rats [95], chlorpromazine-induced Parkinsonism-like signs [96], haloperidol-induced catalepsy [97], and rotenone-induced Parkinson’s disease [98].

### 3.3. Toxicity Studies

Histopathological examinations are usually used to evaluate the nasomucosal toxicities of SLNs and NLCs. After IN administration, fresh nasal mucosa is carefully removed and stored in formalin solution. Samples are stained with hematoxylin and eosin and then examined under a light microscope to detect nasal tissue damage [99]. Biomarkers are also used to investigate toxicity. For example, total protein, lactate dehydrogenase, alkaline phosphatase, and immuno-globulin E levels in nostril fluid are used to study nasomucosal toxicity [99]. Hematological [75] and hepatic biomarkers [100] have also been used in some studies.

## 4. Nose-to-Brain Delivery of SLN and NLC-Based Formulations: Summary of a Literature Search for In Vivo Studies

We searched three electronic databases, including (i) MEDLINE (PubMed), (ii) Scopus, and (iii) Web of Science, for original studies published up to 20 January 2022. The search method is described in Supplementary Material.

**Figure 2:**
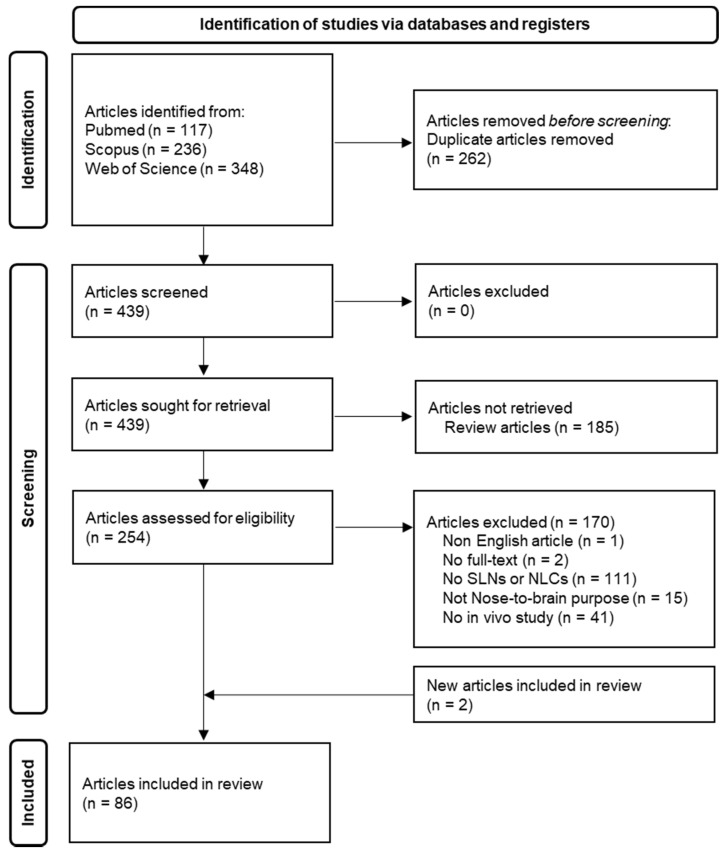
PRISMA 2020 flow diagram explaining the process of searching and screening articles to include in the review.

The processes used to search and screen articles were in accord with PRISMA 2020 guidelines [101]. As shown in Figure 2, after identification and screening steps, 86 articles were included to conduct this systematic review.

Some of these articles performed in vivo studies on the same SLN or NLC formulations, and thus, these articles were based on the results of 81 studies. In detail, the same duloxetine-loaded NLCs were used for two PK and one PD studies in three different articles [73,92,102]. Two articles used haloperidol-loaded NLCs and reported the same PK results [103,104], and an NLC formulation containing resveratrol was used for PK and PD studies in two different reports [85,86]. Two articles used the same olanzapine-loaded NLCs for PK studies in mice [26] and rats [75].

All 86 articles included in this review were published from 2011 to 2021, and half of them were published in the last three years (2019–2021) (Figure S1a). Interest in the nose-to-brain delivery of drugs using SLNs and NLCs and in vivo brain targeting evaluations is increasing. Three types of animals have been used in these studies, including mice, rabbits, and rats. Among them, rat is the most predominant model (Figure S1b). Six studies used two types of animals. SLNs and NLCs were used in suspension form in 48 studies for in vivo evaluation (Figure S1b). Gelling systems and surface modifications were reported in 22 and 11 studies, respectively. NLCs are the second generation of SLNs with more advantages; therefore, 51 studies investigated NLCs and 30 investigated SLNs (Figure S1b). Various production methods were used to prepare SLNs and NLCs in these studies, including high-speed homogenization and ultrasonication, solvent diffusion, solvent evaporation, high-pressure homogenization, solvent injection, microemulsion, and double emulsion. High-speed homogenization and ultrasonication was the most frequently used method in these studies (47%) (Figure S1c). There were 66 PK and biodistribution studies and 30 PD studies. Fifteen studies reported both in vivo PK and PD results (Figure S1d).

**Figure 3:**
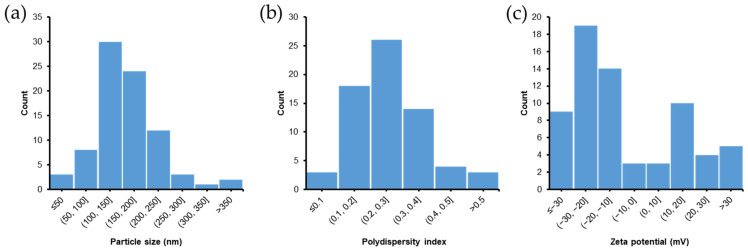
Summary of (**a**) particle size, (**b**) polydispersity index, and (**c**) zeta potential of SLNs and NLCs reported in articles included in the review. *n* = 83 for (**a**), *n* = 68 for (**b**), and *n* = 67 for (**c**).

Reported particle sizes, polydispersity indices, and zeta potentials of SLNs and NLCs are summarized in this review. Particle size is a pivotal factor determining total surface area and physical stability of SLNs and NLCs. As shown in Figure 3a, most of the SLNs and NLCs described had a particle size of <200 nm (78%) and the median particle size was 151.6 nm. Polydispersity index is also a critical parameter, and a value of <0.5 implies a monodispersed and homogenous dispersion of SLNs or NLCs [105]. As shown in Figure 3b, approximately 70% of the SLNs and NLCs had a polydispersity index of <0.3, which is considered an optimum value that indicates a dispersion with a satisfactory size distribution [106]. Three studies reported polydispersity indices of >0.5, indicating non-homogeneity and polydispersity [105]. The median polydispersity index was 0.272.

Zeta potential is the surface charge of a particle, and values were reported in 67 studies with 45 cases of negatively charged and 22 cases of positively charged nanoparticles. Most of the reported zeta potential values (79%) were between −30 and 30 mV (Figure 3c). A dispersion with an absolute zeta potential value of >30 mV is generally considered physically stable. However, in many cases, the stabilities of SLNs and NLCs are maintained by stabilizers even though the zeta potential value was not high [107].

## 5. In Vivo Evaluations of SLN and NLC-Based Formulations for Nose-to-Brain Delivery

This section discusses the efficacy of SLNs and NLCs for nose-to-brain delivery using the in vivo results of previous studies. SLNs and NLCs have been used in suspension forms (plain SLNs and NLCs) or loaded into gels (SLN or NLC gel), and several drugs have been incorporated into SLNs and NLCs to treat a wide range of CNS diseases.

### 5.1. PK Studies with DTE% and DTP% Values

PK and biodistribution evaluations were reported in 66 of the 81 studies. These studies aimed to show SLNs and NLCs (IN) better target the brain than other formulations, such as free drugs (IN, IV, or oral) or IV administered SLNs or NLCs. DTE% and DTP% values were provided in 26 studies. The DTE% and DTP% values were sometimes calculated from raw data when they were not reported in the original articles. When DTE% and DTP% values were incorrectly reported, we recalculated these values. In most cases, DTE% and DTP% values showed SLNs and NLCs (IN) more effectively targeted the brain than free drugs (IN).

Youssef et al. developed SLNs loaded with almotriptan, a second-generation triptan with high selectivity for 5-HT_1B/1D_ receptor used to treat acute migraine [99]. The optimized SLN formulation was dispersed in an in situ nasal mucoadhesive gel composed of Poloxamer 407 and sodium carboxymethyl cellulose (Na-CMC). In rats, SLN gel (IN) exhibited a rapid onset brain targeting (T_max,brain_ = 10 min). DTE% values were 335.7 and 255.1, and DTP% values were 70.21 and 60.80 for SLN gel (IN) and free drug-loaded gel (IN), respectively, which confirmed good targeting efficiencies for both formulations. However, the SLN gel was superior to free drug-loaded gel, as indicated by logRDTE%, logRDTP%, and RB% values of 2.12, 2.06, and 125.9, respectively. In addition, the safety of SLN gel was confirmed through biomarkers’ evaluation and histopathological examination results.

Agomelatine, an antidepressant, was loaded into SLNs for nose-to-brain delivery [67]. The brain targeting efficacy of SLNs (IN) was demonstrated by DTE% and DTP% results of 47.379 and 190.02, respectively. However, the B%_IN/IV_ value was 83.26, which indicated the IN administration of SLNs was not superior to IV administration of the free drug. This research group later loaded the optimized SLNs into an in situ nasal gel composed of 16% Poloxamer 407 and 0.4% HPMC [60]. Interestingly, SLN gel (IN) had lower DTE% (141.42), DTP% (29.29), and B%_IN/IV_ (52.6) values than SLNs (IN). Unfortunately, this issue was not discussed in the original article.

Jain et al. developed NLCs loaded with artemether for the treatment of cerebral malaria [108]. PK studies in rats showed that the brain:blood concentration ratio of NLCs (IN) was higher than those of a drug solution (IN and IV) at all times. DTE% and DTP% values of NLCs (IN) were 278.2 and 64.02, respectively, and logRDTE% (2.07), logRDTP% (2.05), and RB% (254.5) values indicated better brain targeting for NLCs than the drug solution (IN). The B%_IN/IV_ value of 444.8 signified the higher brain bioavailability of the NLCs (IN) over the drug solution (IV).

Asenapine, an antipsychotic drug, was incorporated into NLCs for the treatment of schizophrenia and bipolar disorders via IN administration [28]. In rats, the C_max,brain_ value of NLCs (IN) was 1.4- and 1.8-fold higher than those of IN and IV drug solution, respectively. DTP% values were not reported in this study, and thus we calculated them from raw data. NLCs (IN) showed DTE% and DTP% values of 207.2 and 51.7, respectively, which were higher than those of the drug solution (IN). In addition, B%_IN/IV_ (276.7) and RB% (267.8) values indicated that the brain bioavailability of NLCs (IN) was greater than those of IN and IV drug solution. Moreover, an animal behavioral study using catalepsy-induced rats showed that NLCs (IN) had better therapeutic and safety profiles than the drug solution (IN). Later, this group coated the optimized NLC formulation with glycol CS (GC-NLCs) [29]. In rats, the C_max,brain_ value of GC-NLCs (IN) was found to be further increased (by 1.8- and 2.3-fold higher than those of IN and IV drug solution, respectively). We calculated DTP% from raw data since it was not reported. DTE% (288.3), DTP% (65.31), logRDTE% (2.22), logRDTP% (2.18), B%_IN/IV_ (407.9), and RB% (394.8) values of GC-NLCs (IN) were higher than those for NLCs (IN) in the previous report [28], which indicated that the glycol CS coating increased the brain targeting ability of uncoated NLCs.

Yasir et al. developed donepezil-loaded SLNs for nose-to-brain delivery for the treatment of Alzheimer’s disease [109]. PK studies in rats revealed that C_max,brain_ of SLNs (IN) was 4.1- and 5.4-fold higher than those of IN and IV drug solution, respectively. DTE% and DTP% values of SLNs (IN) were 288.75 and 65.37, higher than those of the drug solution (IN) (156.94 and 36.28, respectively. RB% value of 197.6 showed that SLNs (IN) exhibited better brain targeting than the free drug (IN). In addition, the B%_IN/IV_ value (163.4) indicated that the brain bioavailability of SLNs (IN) was greater than that of drug solution (IV). These results demonstrated the potential merits of using SLNs (IN) to deliver donepezil to the brain.

In another study, donepezil-loaded SLNs produced similar results [110]. The C_max,brain_ of SLNs (IN) was 5.5- and 7.6-fold higher than those of IN and IV drug solution, respectively. The DTE% and DTP% values of SLNs (IN) were 533.95 and 81.94, higher than those of drug solution (IN) (243.78 and 58.84, respectively. Furthermore, RB% (290.9) and B%_IN/IV_ (300.9) values indicated greater brain bioavailability of SLNs (IN) than drug solution (IN or IV). Gamma scintigraphy images of rabbits after IN administration confirmed higher SLNs uptake by brain than drug solution.

Duloxetine, a drug used to treat major depressive disorders, was loaded NLCs for nose-to-brain delivery [92]. NLCs (IN) improved locomotor activity, total swimming, and climbing times, and reduced the duration of immobility period as compared with drug solution (IN or IV). Duloxetine brain concentration for NLCs (IN) was 3.8-fold higher than that for the drug solution (IN). This research group used the same NLC formulation for IN administration in rats and found a 2.39-fold increase in brain concentration for NLCs (IN) as compared with the drug solution (IN) [102]. Later, the authors carried out biodistribution studies in rabbits using ^99m^Tc-labeled duloxetine [73]. The DTE% and DTP% values of NLCs (IN) were 758.1 and 86.81, respectively, which were higher than those of the drug solution (IN) (287.3 and 65.12, respectively). The logRDTE% (2.42), logRDTP% (2.12), and RB% (984.9) values indicated better brain targeting by NLCs than the drug solution (IN). Furthermore, the AUC_0–24h,brain_ of NLCs (IN) was 52.19-fold higher than that of drug solution (IV) (B%_IN/IV_ = 5219), which represented a dramatic improvement in brain bioavailability.

Pokharkar et al. developed NLCs loaded with efavirenz, a potent non-nucleoside reverse transcriptase inhibitor used for the treatment of human immunodeficiency virus (HIV) [65]. IN delivery of the optimized NLCs was employed to target HIV in CNS. PK studies in rats revealed that NLCs (IN) had a 2.5-fold higher C_max,brain_ value than a drug dispersion (IN), while brain bioavailability was ~18-fold higher (RB% = 1782.5). In addition, reported DTE% and DTP% values of NLCs (IN) were 487.4 and 88.2. After recalculation using raw data, these values were 1205 and 91.7; whereas for drug dispersion (IN), they were 48.4 and <0, respectively. In addition, NLCs (IN) remarkably increased brain bioavailability as compared with the pure drug (IV) (B_IN/IV_ = 1272.5%). For the same optimized NLCs, the IN administration showed a 3.73-fold increase in brain bioavailability, as compared with IV administration.

In a recent study, escitalopram and paroxetine were co-loaded into NLCs for the treatment of depression [111]. The optimized NLC formulation was incorporated into a thermoreversible Poloxamer 407 and Carbopol 974P gel. The developed NLC gel was IN administrated to mice, resulting in the different brain targeting efficacies of escitalopram and paroxetine. For paroxetine, the C_max,brain_ of NLC gel (IN) was 4.8- and 5.9-fold higher than those of the free drug (IN and IV, respectively). DTE% and DTP% values of the NLC gel (IN) were 388 and 74.2, respectively, which were also higher than those of the free drug (IN) (232 and 56.9, respectively). B%_IN/IV_ and RB% values (272.5 and 138.3, respectively) showed that the NLC gel (IN) increased brain bioavailability as compared with free paroxetine (IV and IN). Surprisingly, escitalopram had poor brain targeting efficacy (DTE% = 25.4, DTP% = −294, B%_IN/IV_ = 10.5, and RB% = 23.9). The authors suggested that hydrophilic drugs (like escitalopram) were unlikely to exhibit benefits when incorporated into NLC gels (IN). However, several hydrophilic drugs have been successfully delivered to the brain by SLNs and NLCs (IN) [109,110,112]. We found that the drug loadings of escitalopram and paroxetine in NLCs were 1.8 and 4.0%, respectively. This meant it was impossible to use the same doses of these drugs (2.38 mg/kg) in PK studies as was reported. Thus, the dose of escitalopram in NLC gel (IN) was probably lower than that of the free drug (IN and IV) and reported DTE%, DTP%, B%_IN/IV_, and RB% values were incorrect.

Yasir et al. performed PK studies in rats on haloperidol-loaded SLNs (IN) and drug solutions (IN and IV) [103,104]. The drug concentrations in the brain were higher for SLNs (IN) than for drug solution (IN and IV). Notably, C_max,brain_ of SLNs (IN) was 3.7- and 4.3-fold higher than those of drug solutions (IN and IV, respectively). As a result, the brain bioavailability, as determined by AUC_0–24h,brain_, of SLNs (IN) was ~5-fold higher than that of the drug solution (IV) (B%_IN/IV_ = 500.9). DTE% and DTP% values of the drug solution (IN) were 1128.6 and 91.14, respectively. The higher values of SLNs (IN) (DTE% = 2362.4 and DTP% = 95.77) and other parameters (logRDTE% = 2.32, logRDTP% = 2.02, RB% = 349.7) suggested better brain targeting efficiency for SLNs than the drug solution following IN administration.

Levofloxacin and doxycycline were co-loaded into SLNs for meningitis treatment [112]. HPMC gel of the optimized SLNs was prepared to increase drug residence in the nasal cavity. In PK studies using rats, the DTE% (149.8 and 161.9) and DTP% (33.28 and 38.26 (corrected)) of levofloxacin and doxycycline for SLN gel (IN) indicated effective brain targeting. LogRDTE% values (2.19 for levofloxacin and 2.21 for doxycycline) suggested better brain targeting by the SLN gel (IN) over the drug solution (IN). However, the C_max,brain_ and AUC_0–360min,brain_ of levofloxacin and doxycycline of drug solution (IV) were higher than those of the SLN gel (IN). This was attributed to passive diffusion of drugs across the BBB when initial drug concentrations in rat plasma were boosted following IV administration. Thus, although the SLN gel exhibited effective brain targeting, absolute drug accumulations in the brain of the SLN gel (IN) were lower than those of the drug solution (IV). Abourehab et al. developed NLCs loaded with nicergoline for the treatment of dementia [58]. In PK studies using rats, the DTE% value of the NLC gel (IN) (187.3) was based on the AUC of the NLC dispersion (IV), and the DTP% value of the drug solution (IN) was incorrectly reported (180.6). After recalculating using Equations (1) and (2), we found DTE% values of 92.2 and 237.3 and DTP% values of −8.4 and 57.87 for the drug solution (IN) and NLCs (IN), respectively. LogRDTE% (2.41) and RB% (417.6) values indicated better brain targeting by NLCs than the drug solution (IN). The AUC_0–8h,brain_ value of NLCs (IN) was 2.66- and 1.44-fold higher than those of the drug solution (IV) and NLCs (IV), respectively.

Ondansetron was loaded into NLCs for the management of chemotherapy-induced postoperative nausea and vomiting [113]. In PK studies in rats, the C_max,brain_ and AUC_0–330min,brain_ values of the NLCs (IN) were 4.1- and 34.3-fold higher than those of the drug solution (IV), respectively. DTE% and DTP% values of NLCs (IN) were reported to be 506 and 97.14, respectively. However, based on the AUC reported, these values were corrected to 5062 and 98.02, respectively.

Nair et al. developed two types of phenytoin-loaded NLCs with different particle sizes: NLC1 (<50 nm) and NLC2 (>100 nm) [36]. In PK studies using rats, DTE% and DTP% values were not calculated. After calculation from raw data, NLC1 and NLC2 (IN) had DTE% values of ~150,000 and ~72,600 and DTP% values of 99.93 and 99.86, respectively. Thus, the drug had low BBB permeability, and phenytoin predominantly accumulated in brain via direct routes. NLC1 and NLC2 (IN) increased AUC_0–1h,brain_ 48.7- and 36.3-fold (B%_IN/IV_ = 4873 and 3629), respectively, as compared with a phenytoin marketed formulation (IV) and 30.2- and 22.5-fold, respectively, compared with a drug solution (IN).

Uppuluri et al. developed SLNs containing piribedil, an anti-Parkinson’s disease drug, and then loaded optimized SLNs in a thermoresponsive methylcellulose in situ gel [27]. PK studies in rats showed that the SLN gel (IN) and SLN suspension (IN) increased the AUC_0–6h,brain_ by 4- and 3.1-fold (RB% = 404.5 and 312.6), respectively as compared with the drug suspension (IN). DTE% values of the SLN gel (IN) and SLN suspension (IN) were 137.5 and 119.9, respectively, higher than that of the IN drug suspension (54.25). Furthermore, DTP% values (27.29 and 16.59 for SLN gel (IN) and SLN suspension (IN), respectively) revealed efficient direct nose-to-brain delivery, whereas the drug suspension (IN) had a DTP% value below 0.

Risperidone, an atypical antipsychotic, was loaded into SLNs and NLCs in two studies. For risperidone-loaded SLNs, in mouse PK studies, the brain:blood ratio at 1 h of SLNs (IN) was 10- and 5-fold higher than those of drug solution (IV) and SLNs (IV), respectively. The brain targeting of SLNs (IN) was further confirmed by gamma scintigraphy imaging. DTE%, DTP%, and B%_IN/IV_ values for SLNs (IN) were not reported, but calculation from raw data revealed them to be 830.9, 87.97, and 2278.6, respectively, indicating SLNs (IN) achieved effective nose-to-brain delivery. The pharmacodynamic study (paw test with Perspex platform) was conducted using SLNs (IV) and drug solution (IV), but not SLNs (IN) [66].

In another study, risperidone was loaded into CS-coated NLCs [63]. In an in vivo behavioral study using haloperidol-treated rats, CS-NLCs showed greater bio-efficacy with respect to catalepsy and locomotor activity than the drug suspension (IN or IV). Furthermore, in rat PK studies, the C_max,brain_ value of CS-NLCs (IN) was 1.5- and 1.8-fold higher than those of the drug suspension (IN and IV, respectively). DTE% and DTP% values were not reported, but calculation produced DTE% = 252.7 and DTP% = 60.4 for CS-NLCs (IN). They were higher than those of the drug suspension (IN) (logRDTE% = 2.37 and logRDTP% = 2.98), indicating better brain targeting. The brain bioavailability of CS-NLCs (IN) was greater than those of the drug suspension (IN and IV), as evidenced by B%_IN/IV_ = 440.3 and RB% = 308.9.

Gabal et al. developed anionic and cationic NLCs loaded with ropinirole, a drug used to treat Parkinson’s disease [61]. Two types of NLCs were loaded into in situ gels composed of 15% Poloxamer 407, 12% Poloxamer 188, and 1% HPMC. Since ropinirole is a hydrophilic drug with low membrane permeability, its nasal absorption was limited, as evidenced by DTE% = 23.2 and DTP% = −330.2. Following IN administration, the AUC_0–6h,brain_ of the cationic NLC gel was 1.4-fold higher than that of the anionic NLC gel. Cationic and anionic NLC gels both increased the brain bioavailability of ropinirole (RB% = 4087.9 and 5820.3, respectively). C_max,brain_ values were 48- and 81.8-fold higher than that of the drug solution (IN). In addition, both gels had higher half-lives (5- and 8.8-fold, respectively) and mean residence times (MRT) (7.7- and 9.0-fold, respectively) in the brain than a drug solution (IN). DTE% and DTP% values of the anionic NLC gel were 158.5 and 36.9, respectively, higher than those of the cationic NLC gel (128.6 and 22.3, respectively). However, the B%_IN/IV_ values of both NLC gels were <100, indicated that the absolute brain bioavailability of NLC gels (IN) was lower than that of the drug solution (IV).

Sesamol, a potential candidate for the treatment of glial cancer, was incorporated into SLNs for nose-to-brain delivery [114]. SLNs (IN) had a shorter T_max,brain_ (10 min versus 30 min) and a higher C_max,brain_ (13.2-fold) than the free drug (IV). DTE% (764), DTP% (86.1), and B%_IN/IV_ (590.4) values indicated effective brain targeting by the SLNs (IN) over the free drug (IV).

Masjedi et al. developed NLCs loaded with sumatriptan, a selective 5-HT_1B_ and 5-HT_1D_ receptor agonist used for relieving migraine and cluster headache [115]. PK studies in rats showed that the C_max,brain_ of the NLCs (IN) was 5.6-, 7.3-, and 9.4-fold higher than those of drug solution (IN), drug solution (IV), and NLCs (IV), respectively. The authors reported DTE% = 258 and DTP% = 61.23% for NLCs (IN), but these values were based on a comparison with NLCs (IV). When recalculated using Equations (1) and (2), actual values were 2416 and 95.86, respectively. The logRDTE% (2.60), logRDTP% (2.06), and RB% (744.6) values indicated better brain targeting for the NLCs than the drug solution (IN). Furthermore, the AUC_0–4h,brain_ value of the NLCs (IN) was 12.95- and 7.70-fold higher than those of the drug solution (IV) and NLCs (IV), respectively.

Tarenflurbil, a potential candidate for treating Alzheimer’s disease, was loaded into SLNs for nose-to-brain delivery [68]. In rat PK studies, after a single dose, C_max,brain_ for SLNs (IN) was 1.5-, 1.7-, and 4.1-fold higher than those for drug solution (IV), drug solution (IN), and drug suspension (oral), respectively. DTE% and DTP% values were 183.2 and 45.4, respectively. LogRDTE% (2.20), logRDTP% (2.55), and RB% (182.1) indicated higher brain targeting for SLNs (IN) than the drug solution (IN). Furthermore, the brain bioavailability of SLNs (IN) was 1.42- and 3.83-fold higher than those of the drug solution (IV) and drug suspension (oral). In a multiple-dose study, drug concentrations in the brain after 5 and 10 days for SLNs (IN) were approximately 2-fold higher than those for the drug solution (IV) and drug suspension (oral).

Khan et al. prepared NLCs loaded with temozolomide, an effective antineoplastic drug used to treat metastatic melanoma and glioma [116]. In rat PK studies, the optimized NLCs (IN) had a DTE% value of 457, which was ~4-fold higher than that of the drug dispersion (IN). However, the reported DTE% value for the drug dispersion (IN) was incorrect (169.7%), and the corrected value was 113.3. DTP% values were not reported. When calculated from the raw data, DTP%, logRDTE%, logRDTP%, B%_IN/IV_, and RB% values of the NLCs (IN) were 78.16, 2.61, 2.82, 588.1, and 282.7, respectively, indicating effective brain targeting by IN administration of the NLCs. The accumulation of NLCs in the brain was confirmed using gamma scintigraphy images.

Sarma et al. developed NLCs loaded with tenofovir for the treatment of HIV in the brain [78]. In rat PK studies, optimized NLCs had a 3.2, 5.8, and 6.5-fold higher C_max,brain_ than NLCs (IV), drug solution (IV), and drug solution (IN), respectively. DTE% and DTP% values were not reported, and calculation from raw data produced values of 481.9 and 79.25, respectively for the NLCs (IV). LogRDTE% (2.22), logRDTP% (2.08), and RB% (402.7) values indicated higher brain targeting for the NLCs than the drug solution (IN). Furthermore, the brain bioavailability of NLCs (IN) was 12.0- and 3.6-fold higher than those of drug solution (IV) and NLCs (IV), respectively. Confocal microscopic images of rat brain tissue showed coumarin 6-labeled NLCs accumulated until 24 h after IN administration, whereas accumulation following IV administration was negligible for the same formulation.

Ziprasidone was loaded into NLCs for the management of schizophrenia via nose-to-brain delivery [117]. In rat PK studies, NLCs (IN) showed higher brain:blood concentration ratios at all time points and a faster onset (10 min) than drug solution (IV). DTE% value was 476.8, but DTP% value was incorrectly reported (89.85%). Recalculation from raw data produced DTP% = 79.0, indicating brain targeting by NLCs (IN).

Major features of SLN and NLC-based formulations for nose-to-brain delivery in these studies are summarized in Table 1.

### 5.2. PK and Biodistribution Studies without DTE% and DTP% Values

In the remaining 40 PK and biodistribution studies, DTE% and DTP% values were unavailable and thus, the brain targeting efficiencies of SLNs and NLCs (IN) were evaluated using other parameters.

#### 5.2.1. Comparisons Using Brain Bioavailability

In some studies, the brain bioavailabilities of SLNs and NLCs (IN) were compared with free drug solution (IN, IV, or oral) or with SLNs and NLCs (IV). For example, the AUC_0–8h,brain_ of almotriptan-loaded CS-coated NLCs (CS-NLCs) following IN administration to rabbits was 4.56- and 8.06-fold higher than those of Migrostop tablets (oral) and drug solution (IN), respectively [70]. C_max,brain_ of CS-NLCs (IN) was ~7.3 and 7.6-fold higher than those of Migrostop tablets (oral) and drug solution (IN), respectively. T_max,brain_ was observed at 10 min for CS-NLCs (IN), whereas values for Migrostop tablets (oral) and drug solution (IN) were 60 and 20 min, respectively. The brain:blood concentration ratios of CS-NLCs (IN) were in the range 1.4–2.2, whereas the ranges of Migrostop tablet (oral) and drug solution (IN) were 0.41–0.89 and 1.0–1.92, respectively. These results indicated effective brain targeting by CS-NLCs (IN).

Singh et al. developed SLNs loaded with alprazolam, an antianxiety drug [38]. SLNs and drug solution were labeled with ^99m^Tc for biodistribution studies using rats. DTE% and DTP% values of SLNs (IN) and drug solution (IN) were based on the AUC of SLNs (IV). Recalculation was not possible because there was no drug solution (IV) group. SLNs (IN) had higher DTE% and DTP% values than the drug solution (IN). Likewise, AUC_0–8h,brain_ of SLNs (IN) was 2- and 1.33-fold higher than those of drug solution (IN) and SLNs (IV), respectively. Biodistribution studies also revealed that the IN route caused less drug accumulation in liver, spleen, intestine, and kidney than the IV route. Scintigraphy images taken in rabbits revealed more drug deposition in the brain for SLNs (IN) than for SLNs (IV).

In a recent study, astaxanthin-loaded NLCs were developed to improve the treatment of Parkinson’s disease [97]. The optimized NLC formulation was incorporated in an in situ gel composed of 20% Poloxamer 407 and 0.5% CS. In rats, the NLC gel (IN) had higher C_max,brain_ (9.5-fold) and AUC_brain_ (7.79-fold) values than the free drug gel (IN). The reported DTP% value of 99.75 was calculated using data for NLCs (IV). In haloperidol-treated rats, the NLC gel (IN) improved rat behaviors in the rotarod test and akinesia measurements as compared with the free drug gel (IN).

Buspirone, an anxiolytic agent, was loaded into SLNs [77]. In rats, the C_max,brain_ of SLNs (IN) was 1.7- and 2.3-fold higher than those of drug solution (IN) and SLNs (IV). DTE% (882.6) and DTP% (88.67) values were based on the AUC of SLNs (IV). This research group later developed CS-coated NLCs loaded with buspirone [118]. CS-NLCs (IN) had higher C_max,brain_ (1.5- and 2.6-fold) and AUC_0–12h,brain_ (2.2- and 3.1-fold) values than the drug solution (IN) and NLCs (IV), respectively. DTE% and DTP% values of CS-NLCs (IN) were 1462.5 and 93.16, respectively, which were higher than those of the drug solution (IN) (544.4 and 81.63, respectively). However, these values were based on the AUC of CS-NLCs (IV).

Tripathi et al. developed cinnarizine-loaded NLCs and incorporated them in in situ gel for the treatment of migraine [119]. In rats, the optimized NLC gel (IN) showed higher C_max,brain_ (2.07-fold) and AUC_0–4h,brain_ (2.23-fold) values than the drug solution (IN). In formalin-induced acute nociception rat model, the NLC gel (IN) exhibited higher antinociceptive activity for neurogenic pain and inflammatory pain than the free drug solution (IN). In a recent study, clozapine-loaded NLCs were developed to enhance the treatment of schizophrenia [69]. In mice, the C_max,brain_ and AUC_0–12h,brain_ values of NLCs (IN) were 11.8- and 6.15-fold higher than those of clozapine tablets (oral). Curcumin was loaded into NLCs to target brain tumors via IN administration [120]. In rats, C_max,brain_ and AUC_0–48h,brain_ values of NLCs (IN) were 1.6- and 2.2-fold higher than those of drug suspension (IN), respectively.

Butani et al. developed donepezil-loaded NLCs and incorporated the optimized NLCs into ionic-triggered gellan gum matrix for IN delivery [87]. In rats, the AUC_0–8h,brain_ of the NLC gel (IN) was 1.26-fold higher than that of a tablet (oral). This slight increase indicated that the NLC gel was not highly potential for brain targeting. No PK data were provided for IV or IN administrations of the free drug. In a rat model of scopolamine-induced amnesia, the NLC gel (IN) improved cognitive function as compared with a marketed tablet (oral). Recently, flibanserin-loaded NLCs were developed for the treatment of hypoactive sexual desire disorder in premenopausal women [121]. The optimized NLC formulation was incorporated into in situ gel of 0.6% gellan gum. In rats, the NLC gel (IN) had higher C_max,brain_ (3.5-fold) and AUC_0-inf,brain_ (6.3-fold) values than the flibanserin gel (IN). Lurasidone-loaded NLCs were developed to improve the treatment of schizophrenia and bipolar disorder [122]. In rats, NLCs (IN) had higher C_max,brain_ (1.9- and 7.9-fold) and AUC_0–24h,brain_ (2.96- and 9.3-fold) values than the drug solution (IN) and drug suspension (oral), respectively.

Gadhave et al. developed olanzapine-loaded NLCs and incorporated the optimized NLCs into Poloxamer 407-HPMC gel [26]. PK studies were performed using NLC gel and NLC dispersion with radiolabeled olanzapine (^99m^Tc). C_max,brain_ and AUC_0–6h,brain_ values of NLC gel (IN) were 3.98- and 3.81-fold higher than those of the NLC dispersion (IV). The same group later reported that this NLC gel had DTE% and DTP% values of 54,550 and 99.81, respectively [75], but these values were based on AUC of NLCs (IV). Notably, the NLC gel (IN) showed higher brain bioavailability than a microemulsion gel (IN) and NLCs (IV). Furthermore, the NLC gel did not show any evidence of hematological or liver toxicity following IN administration.

Palagati et al. developed oleuropein-loaded NLCs for the treatment of meningitis [123]. In rats, the AUC_0–6h,brain_ of the optimized NLCs (IN) was 2.23-fold higher than that of the NLCs (IV). Although a DTP% value of 83.07 was reported, data was insufficient to enable recalculation. In a previous study, borneol-stearic acid (Bo-SA) conjugate was prepared to enhance the brain targeting of Pueraria flavones-loaded SLNs [124]. In rats, the AUC_0–8h,brain_ and C_max,brain_ values of Bo-SA-SLNs (IN) were 8.31- and 8.29-fold higher than those of SLNs (IN), respectively.

Sivadasu et al. developed NLCs loaded with quetiapine, one of the most effective schizophrenia drugs [125]. IN administration of the optimized NLCs showed 4.15- and 3.57-fold increases in the C_max,brain_ and AUC_0–6h,brain_ values, respectively, as compared with IV administration. The authors also reported DTE% and DTP% values of 485.76 and 90.26, respectively. However, these values were based on AUC of NLCs (IV). A previous study reported the development of a gellan gum and xanthan gum in situ gel containing resveratrol-loaded NLCs for the treatment of Alzheimer’s disease [85]. PD studies (Morris Water Maze test) of a scopolamine-induced amnesia rat model showed that the NLC gel (IN) significantly improved memory function compared with a drug suspension (oral). This group later used the same gel to perform PK studies in rats [86]. The NLC gel (IN) showed higher C_max,brain_ (2.6-fold) and AUC_0–8h,brain_ (1.4-fold) values than the drug suspension (oral).

Rizatriptan-loaded SLNs were developed to improve the management of migraine [126]. In rats, SLNs (IN) showed higher C_max,cerebrospinal fluid_ (1.3- and 5.5-fold) and AUC_0-inf,cerebrospinal fluid_ (1.7- and 3.0-fold) values than the drug solution (IV) and a marketed tablet (oral), respectively. Based on AUC_0-inf,cerebrospinal fluid_, DTE% and DTP% values for the SLNs (IN) were 263.6 and 62.1, respectively. Recently, Pardeshi et al. incorporated ropinirole-dextran sulfate conjugation into NLCs, which were then coated with N,N,N-trimethyl CS [127]. In mice, NLCs (IN) had a higher C_max,brain_ than NLCs (IV) and drug conjugate solution (IN) (1.7- and 17.4-fold, respectively). No PK data were provided for the drug conjugate solution (IV) to enable DTE% and DTP% calculations. Nevertheless, the brain targeting of NLCs (IN) was confirmed based on brain bioavailability as the NLCs (IN) had a higher AUC_0–12h,brain_ value than NLCs (IV) or drug conjugate solution (IN) (1.5- and 13.7-fold, respectively).

Kumar et al. developed SLNs loaded with streptomycin to treat cerebral tuberculosis [72]. In vivo studies were carried out using ^99m^Tc-labeled streptomycin. In a mouse biodistribution study, the brain concentrations of streptomycin for SLNs (IN) were higher than those for the drug solution (IN) (4.57-fold at 0.5 h and 6.0-fold at 24 h). Likewise, AUC_0-inf,brain_ was 3.6-fold higher for SLNs (IN). Gamma scintigraphic images of rabbits confirmed the higher deposition of ^99m^Tc-labeled streptomycin in the brain for SLNs (IN).

In a recent study, ^99m^Tc radiolabeled teriflunomide was loaded into NLC gellan gum-carbopol 974P gel [74]. In mice, the brain:blood concentration ratios of the NLC gel (IN) were approximately 2–3- and 8–10-fold higher than those of the NLC dispersion (IN and IV, respectively). DTE% values of the NLC gel (IN) and NLC dispersion (IN) (1500 and 92, respectively) were based on the AUC for the NLC dispersion (IV). DTP% values of the NLC gel (IN) and NLC dispersion (IN) were incorrectly reported (283 and 42.5, respectively), whereas they should have been 93.3 and −8.7, respectively, based on the AUC for the NLC dispersion (IV). The AUC_0-inf,brain_ of the NLC gel (IN) was 1.34-fold higher than that of the NLC dispersion (IV), whereas the AUC_0-inf,brain_ of the NLC dispersion (IN) was 2-fold lower than that of the NLC dispersion (IV). Thus, the incorporation of NLCs into gellan gum-carbopol 974P gel critically increased brain targeting.

Table 2 summarizes major features of SLN and NLC-based formulations for nose-to-brain delivery in these studies.

#### 5.2.2. Comparison Using Brain: Blood Concentration Ratios

In some cases, brain bioavailability was unavailable, and thus, brain:blood concentration ratios were used instead. For example, Gupte et al. developed efavirenz-loaded SLNs. The brain:blood concentration ratio at 24 h in rats of SLNs (IN) was approximately 150-fold higher than that of a marketed tablet (oral) [128]. Pioglitazone, a drug used to manage Alzheimer’s disease, was incorporated into NLCs for IN administration [129]. In in vivo biodistribution studies using rats, the optimized NLCs (IN) had a 1.9- and 10.7-fold higher brain:blood concentration ratio than drug solution (IN and IV, respectively).

Rimonabant, a cannabinoid antagonist used to treat many CNS diseases, was loaded into NLCs [130]. In rats, the brain:blood concentration ratio at 6 h after administration of NLCs (IN) was 17.11, higher than that of the drug solution (IN) (11.74). However, we found that drug concentration in the brain at 6 h was 5.8 µg/g for the NLCs (IN), which was slightly higher than that (5.1 µg/g) of the drug solution (IN). Eskandari et al. prepared NLCs loaded with valproic acid, a drug widely used to treat migraine, bipolar disorder, epilepsy, and cancer [80]. In rats, the brain:plasma concentration ratio at 60 min after administration was 8.4 for optimized NLCs (IN), which was 5.09-fold higher than that for NLCs (intraperitoneal, IP). In addition, NLCs (IN) provided the same protective effect against seizure in rats (maximal electroshock seizure model) as IP administration of the drug solution, but at a 37.5-fold lower dose.

Table 3 summarizes the major features of SLN and NLC-based formulations for nose-to-brain delivery in these studies.

#### 5.2.3. Drug Accumulation in the Brain

Some studies used drug concentrations in the brain for comparison purposes. For example, artemether and lumefantrine were co-loaded in N,N,N-trimethyl chitosan-coated NLCs (TMC-NLCs) for the treatment of cerebral malaria [64]. In mice, TMC-NLCs (IN) produced higher drug concentrations in the brain than the drug suspension (IN and oral). In parasite-infected mice, parasite suppression on day 7 for TMC-NLCs (IN) was 95% higher than that for IN NLCs (82.5%), IN drug suspension (79.1%), and oral drug suspension (46.3%). In another study, ^99m^Tc radiolabeled astaxanthin-loaded SLNs were developed for the management of neurological disorders [71]. SLNs (IN) achieved ~2-fold higher drug concentrations in brain than IV administration (at 1 h). This finding was confirmed by gamma scintigraphy imaging.

Esposito et al. developed SLNs loaded with dimethyl fumarate for the treatment of multiple sclerosis [131]. SLNs were labeled with indocyanine green for fluorescent luminescent imaging. SLNs (IN) showed the brain accumulation similar to that of SLNs (IP), but at a 10-fold lower dose. A previous study reported the development of NLCs loaded with embelin for the treatment of epilepsy [82]. Optimized NLCs (IN) resulted in higher drug concentrations in the brain than the drug solution (IN) and a marketed formulation (IV). However, there was insufficient data to calculate DTE% and DTP% values. In pentylenetetrazole-treated rats, NLCs (IN) reduced malondialdehyde and nitrite levels and increased glutathione levels as compared with drug solution (IN) and a marketed formulation (IV).

Saini et al. developed CS-coated SLNs containing ferulic acid for the treatment of Alzheimer’s disease [90]. The drug concentrations in rat brain for CS-SLNs (IN) and SLNs (IN) were 6.91- and 5.42-fold higher than that for a drug suspension (IN), respectively. In rat model of Alzheimer’s disease (induced by streptozocin), both CS-SLNs (IN) and SLNs (IN) improved cognitive ability and biochemical parameters (lipid peroxidation, nitrite, superoxide dismutase, acetylcholinesterase, glutathione, and protein levels in cortex and hippocampus) as compared with a drug suspension (IN, oral) and SLNs (oral). CS-SLNs (IN) were more effective than SLNs (IN).

Lamotrigine-loaded NLCs were developed to improve the treatment of epilepsy [81]. Gamma scintigraphy studies showed that drug accumulated in the brain until 6 h after IN administration of NLCs. At 24 h after administrations, drug concentration in the brain for NLCs (IN) was 1.4- and 5.1-fold higher than those for drug solutions (IN and oral, respectively). Likewise, in rats with seizures, NLCs (IN) improved behavioral abnormalities, decreased malondialdehyde, and increased glutathione in the brain as compared with drug solution (IN and oral). Hasan et al. developed SLNs loaded with naloxone, an opioid receptor antagonist used to treat opioid overdose [132]. The drug was radiolabeled with ^99m^Tc, and gamma scintigraphy and biodistribution studies in rats showed better deposition of naloxone in the brain for SLNs (IN) than for drug solution (IN). The PK and biodistribution studies were conducted in rabbits on only SLNs (IN), and thus, DTE% and DTP% values could not be calculated.

Sun et al. developed a paeonol-loaded SLN gel (0.4% deacetylated gellan gum and 0.3% HPMC) [12]. The authors found that brain accumulation was better for a cyanine7 NHS ester-loaded SLN gel (IN) than cyanine7 NHS ester-loaded SLNs (IV). Li et al. prepared a quetiapine-loaded SLN gel and evaluated it in a rat model of schizophrenia [133]. It was found that drug concentrations in the prefrontal cortex, cerebellum, hippocampus, and pituitary after SLN gel (IN) administration were similar to those of the drug solution (IV) and significantly higher than those of the drug solution (oral) at all time points. Furthermore, the SLN gel (IN) improved hippocampal morphology more than drug solution (IV and oral) in a rat schizophrenia model.

Wavikar et al. developed rivastigmine-loaded NLCs for the treatment of Alzheimer’s disease [88]. The optimized NLC formulation was incorporated into an in situ gel (15% Poloxamer 407 and 0.8% gellan gum). Biodistribution studies in rats showed 4.6-, 8.6-, and 1.6-fold higher drug concentration in the brain at 1 h for the NLCs gel (IN) than for the drug solution (IN), drug solution (IV), and NLCs (IV), respectively. Furthermore, in scopolamine-induced amnesic mice, the NLC gel (IN) exhibited faster regain of memory loss than the drug solution (IN and IV).

Some studies reported drug accumulations in the brain for SLNs and NLCs (IN) without using a reference (control) formulation, which was insufficient to demonstrate brain targeting efficiencies as compared with other formulations. For example, when NLCs loaded with a geraniol-ursodeoxycholic acid conjugate were developed for the management of Parkinson’s disease [134], NLCs (IN) were found to deliver drug from the nose to the brain in rats without causing mucosal irritation, and the drug was detected in cerebrospinal fluid until 3 h after administration. However, the authors did not perform PK studies on drug solutions (IN and IV) for comparison. Gartziandia et al. developed CS-coated NLCs containing human insulin-like growth factor-I (hIGF-I)) [76]. Fluorescence imaging revealed that DiR DiIC18-labeled CS-NLCs presented in the nasal cavity until 24 h and rapidly distributed in the olfactory tract and brain after IN administration. However, no other formulations (e.g., IV and IN drug solutions) were examined.

Khanna et al. developed SLNs loaded with nalbuphine for the management of pain [135]. SLNs were radiolabeled with ^99m^Tc. Biodistribution and gamma scintigraphy studies revealed their presence in the brain 10 min after administration and their retention until 8 h. SLNs (IN) produced better analgesic effects and had early action onsets than the drug solution (intramuscular injection) in thermal allodynia-induced rats. In another study, the ^99m^Tc-SLNs (IN) loaded with ondansetron exhibited rapid accumulation (1 h post-administration) in rabbit brains as determined by gamma scintigraphy [136].

Bhatt et al. developed SLNs loaded with rosmarinic acid for the management of Huntington’s disease [137]. Rat PK studies showed drug accumulation amount in the brain of 5.69 µg; but unfortunately, no detailed data were provided. In 3-nitropropionic acid-treated rats, SLNs (IN) improved behavioral abnormalities and attenuated oxidative stress (by decreasing malondialdehyde and nitrite levels and increasing catalase and glutathione levels in the brain) as compared with SLNs (IV) or free rosmarinic acid (IN). In a recent study, zolmitriptan-loaded SLNs were incorporated into HPMC gel [138]. Histopathological examination of brain tissues showed that SLNs accumulated in the brain cells until 24 h after IN administration.

Major features of SLN and NLC-based formulations for nose-to-brain delivery in these studies are summarized in Table 4.

### 5.3. PD Studies

Among the 30 studies that reported PD evaluations, 15 performed PK or biodistribution studies and these have been mentioned above. The remaining 15 articles were as follows.

Agbo et al. developed NLCs loaded with artesunate for the treatment of severe cerebral malaria [57]. In mice infected with *Plasmodium berghei* ANKA, activity and parasitemia reduction for NLCs (IN) (54.70 and 33.28%, respectively) were comparable to those for intramuscular administration (58.80 and 42.18%, respectively). These results suggested that artesunate-loaded NLCs (IN) might be a satisfactory alternative to conventional intramuscular administration, which can be problematic in remote areas. In a recent study, Matarazzo et al. developed NLCs containing cannabidiol, a phytocannabinoid used to treat chemotherapy-induced peripheral neuropathy in cancer patients [139]. Cetylpyridinium chloride was used as a surfactant to produce positively charged NLCs. In mice with paclitaxel-induced neuropathic pain, the NLC dispersion (IN) had greater antinociceptive effects than the drug solution (IN and oral).

Deshkar et al. developed NLCs containing carbamazepine, an anticonvulsant and antiepileptic [79]. The NLCs were loaded into in situ gel (20% Poloxamer 407, 5% Poloxamer 188, and 0.2% CS). The NLC gel (IN) improved in vivo anticonvulsant activity in rats with maximal electroshock seizure as compared with a carbamazepine dispersion (oral) or carbamazepine in situ gel (IN). In a previous study, superparamagnetic iron oxide-loaded NLCs (SPION-NLCs) containing clonazepam were developed for the treatment of epilepsy [83]. SPION was used to increase drug targeting with the help of an external magnetic field. The optimized formulation was incorporated into in situ gel (15% Poloxamer 407 and 0.75% sodium alginate). In pentylenetetrazole-induced convulsion mouse model, the SPION-NLC gel (IN) and NLC gel (IN) prolonged the onset of convulsion (7.5- and 1.5-fold) and death (14- and 5-fold), respectively, as compared with the non-treated controls.

Vitorino et al. developed NLCs loaded with fluoxetine for the treatment of depression [93], and examined its effects in mice using marble-burying and forced swimming tests. The results obtained showed that NLCs (IN) had more potent anti-depressive and anxiolytic effects than the drug solution (oral). Gartziandia et al. loaded glial cell-derived neurotrophic factor (GDNF) into CS-NLCs for nose-to-brain delivery [95]. In 6-hydroxydopamine partially lesioned rats, IN administration of CS-NLCs induced greater behavioral improvements and had more potent neuroprotective and neurorestorative effects than the oral drug solution. Hernando et al. also incorporated GDNF into NLCs coated with CS (CS-NLCs) or a transactivator of transcription (TAT) peptide-CS conjugate (TAT-CS-NLCs) [94]. In a 1-methyl-4-phenyl-1,2,3,6-tetrahydropyridine-treated mouse model of Parkinson’s disease, CS-NLCs (IN) and TAT-CS-NLCs (IN) improved motor recovery and increased numbers of tyrosine hydroxylase fibers in striatum and tyrosine hydroxylase neuron levels in substantia nigra more than GDNF solution (IN).

Ketononazole was loaded into NLCs to improve the treatment of cryptococcal meningoencephalitis [140]. In mice infected with fungal cells, NLCs (IN) reduced the fungal burden in the brain more than free ketononazole (IN). Taymouri et al. developed NLCs containing lorazepam, a drug used to treat epilepsy [84]. The optimized NLC formulation was loaded into an in situ gel of CS and β-glycerol phosphate for nose-to-brain delivery. In pentylenetetrazole-treated rats, the NLC gel (IN) reduced seizure occurrence versus the NLC dispersion (IN) and lorazepam solution (IP). A previous study reported the development of NLCs loaded with rivastigmine for the treatment of dementia [89]. The optimized NLCs (IN) showed noticeable improvements in escape and transfer latencies (using the Morris water maze test and the elevated plus maze test) in scopolamine-treated rats as compared with drug solution (IN).

Pardeshi et al. developed SLNs loaded with ropinirole [96]. In chlorpromazine-treated mice exhibiting Parkinsonism-like signs, the optimized SLNs (IN) showed better anti-tremor activity than a marketed tablet (oral) at a 3.3-fold lower dose. Another study reported the development of NLCs loaded with selegiline for the treatment of Parkinson’s disease [98]. In rotenone-treated rats, the NLCs (IN) restored behavior and malondialdehyde, nitrite, and glutathione levels better than free selegiline (IN). Hangargekar et al. developed SLNs loaded with sertraline and evaluated the antidepressant effects of the optimized SLNs on rats using the tail suspension and forced swimming tests [91]. SLN (IN) and the free drug (IN) reduced immobility duration in both tests, but SLN (IN) did so at a 2.5-fold higher dose.

Gadhave et al. developed NLCs loaded with teriflunomide for the treatment of multiple sclerosis [100]. The optimized NLC formulation was incorporated into in situ gel (17% Poloxamer 407 and 0.3% HPMC). In a cuprizone-induced rat model of microglia activation and demyelination, the NLC gel (IN) induced more rapid remyelination and behavior improvements than NLCs (oral). Esposito et al. developed SLNs loaded with URB597, an endocannabinoid hydrolysis inhibitor, for the treatment of depression [141]. The SLNs (IN) induced behavioral effects similar to those induced by URB597 solution (IP).

Table 5 summarizes the major features of SLN and NLC-based formulations for nose-to-brain delivery in the 30 PD studies.

### 5.4. Toxicity Studies

Toxicity studies were performed in 30 of the studies. In most cases, SLN and NLC-based formulations were reported to be safe, as demonstrated by histopathological and biomarker examinations. For example, in rats, an almotriptan-SLN gel did not show any signs of mucosal damage, cilia loss, or cell necrosis, while total protein, lactate dehydrogenase, alkaline phosphatase, or immunoglobulin E in notril lavage fluids were in safe levels [99]. An in situ Poloxamer 407-HPMC gel of olanzapine-loaded NLCs did not increase the risk of hematological or liver toxicity following IN administration [75]. In another study, teriflunomide-loaded NLCs reduced the risk of hepatotoxicity as compared with the free drug [100]. However, one study reported destruction of nasomucosal epithelium after the IN administration of cationic ropinirole-loaded NLCs to rats. Fortunately, the nasal irritation and inflammation disappeared when NLCs were incorporated into Poloxamer thermosensitive in situ gels [61].

## 6. Evaluation of PK Parameters for Nose-to-Brain Delivery

We analyzed the PK parameters collected from studies included in this review, including DTE%, DTP%, logRDTE%, logRDTP%, B%_IN/IV_, RB%, and AUC_brain_/AUC_blood_ ratios. Statistical analysis was performed using R, version 3.6.1. The normality of variables distribution was evaluated using the Shapiro-Wilk’s normality test. Differences between mean logDTE%, DTP%, and logB%_IN/IV_ values of free drugs (IN) and SLN and NLC-based formulations (IN) were assessed using the *t*-test (for normal distributions). Differences between mean AUC_brain_/AUC_blood_ ratios of free drugs (IN), SLN and NLC-based formulations (IN), and free drugs (IV) were assessed by one-way analysis of variance (ANOVA) with a Tukey’s Honestly Significant Difference (Tukey’s HSD) post-hoc test for pairwise comparisons.

**Figure 4:**
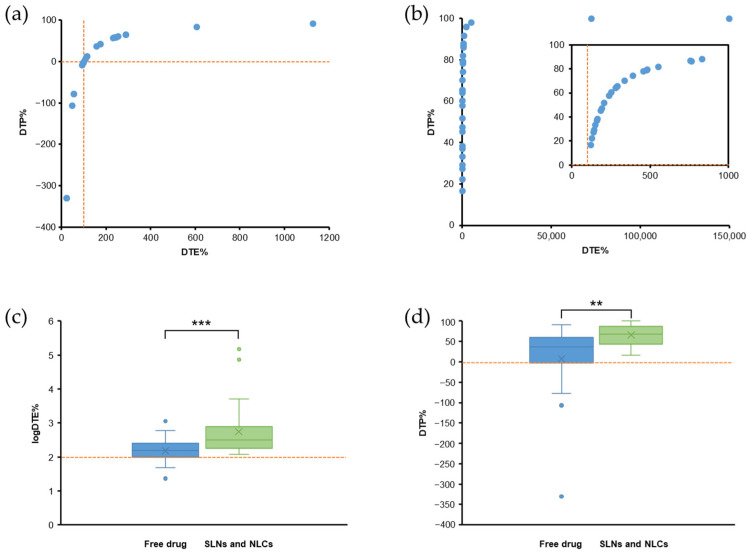
Summary of DTE% and DTP% for (**a**) free drug (IN) and (**b**) SLN and NLC-based formulation (IN). The inset in (**b**) shows data with DTE% < 1000. The vertical dot-lines in (**a**,**b**) are for DTE% = 100; the horizontal dot-line in (**a**) is for DTP% = 0. (**c**,**d**) Comparison of logDTE% and DTP% between free drug (IN) and SLN and NLC-based formulation (IN) using boxplots. × indicates mean value. The horizontal dot-lines in (**c**) and (**d**) are for logDTE% = 2 and DTP% = 0, respectively. *n* = 21 for free drug (IN) and *n* = 30 for SLN and NLC-based formulation (IN). ** *p* < 0.01 and *** *p* < 0.001 (*t*-test).

**Figure 5:**
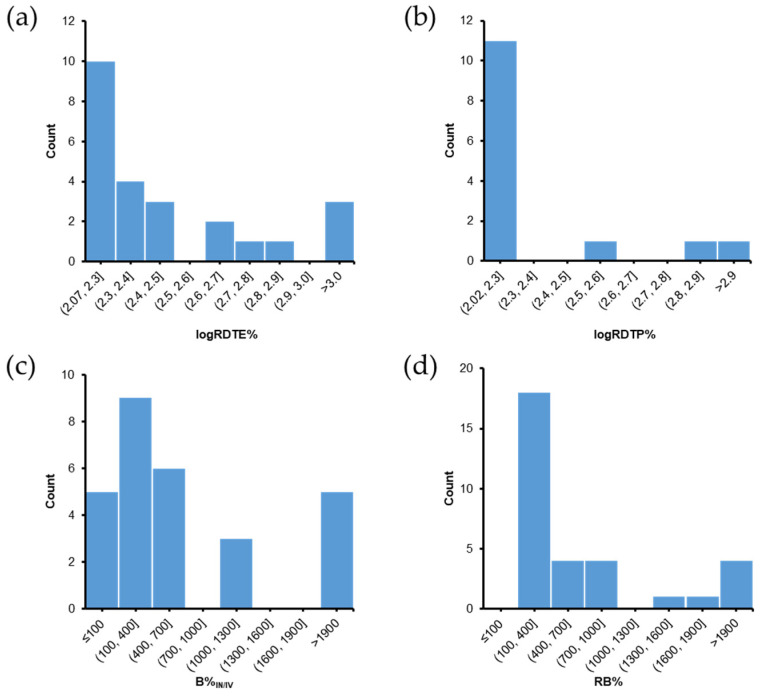
Summary of (**a**) logRDTE% (*n* = 24), (**b**) logRDTP% (*n* = 14), (**c**) B%_IN/IV_ (*n* = 28), and (**d**) RB% (*n* = 32) for SLN and NLC-based formulation.

The DTE% and DTP% values of free drugs (IN) and SLN and NLC-based formulations (IN) (Figure 4a,b) showed perfect correlations, as expressed by Equation (3). For free drugs (IN), 33.3% of studies had DTE% values of <100 and DTP% values of <0 (Figure 4a), indicating insufficient brain targeting. It may have been due to some limitations of the IN administration [15,16]. All SLN and NLC-based formulations had DTE% values > 100 and DTP% values > 0 (Figure 4b). Furthermore, mean values of logDTE% and DTP% (2.74 and 65.61, respectively) for SLN and NLC-based formulations were significantly greater than those of free drugs (2.18 and 7.72, respectively) (Figure 4c,d). When DTE% and DTP% values of SLN and NLC-based formulations (IN) and free drugs (IN) were compared in same studies, all logRDTE% and logRDTP% values were >2 (Figure 5a,b). Mean values of logRDTE% and logRDTP% were 2.61 and 2.25, respectively. These results show that SLN and NLC-based formulations (IN) improved brain targeting of drugs.

However, B%_IN/IV_ and RB% values are also important when evaluating the efficacies of SLN and NLC-based formulation (IN). Figure 5c shows that B%_IN/IV_ values were <100 in 5 of 27 reported cases. They are agomelatine-loaded SLNs [67], agomelatine-loaded SLN gel [60], levofloxacin and doxycycline-loaded SLNs [112], ropinirole-loaded anionic and cationic NLCs [61]. In these cases, although DTE% (>100) and DTP% (>0) values suggested effective brain targeting of SLN and NLC-based formulation (IN), B%_IN/IV_ values indicated that absolute brain bioavailabilities of SLN and NLC-based formulation (IN) were lower than those of the free drug (IV). Thus, in these cases, the therapeutic effects of SLN and NLC-based formulations (IN) are probably not greater than those of free drugs (IV). Low B%_IN/IV_ values could be due to substantial sample losses in the throat and gastrointestinal tract [66,116], which would reduce AUC_brain_ and AUC_blood_ values of SLN and NLC-based formulations (IN). Median and mean B%_IN/IV_ values were 354.4 and 1017.0, respectively. The highest B%_IN/IV_ was 5219.2. B%_IN/IV_ values were >1900 in 5 cases, which had DTE% values of >750 and DTP% values of >86. Figure 5d shows that RB% values were >100 for all reported cases, indicating higher absolute brain bioavailabilities for SLN and NLC-based formulations (IN) than for free drugs (IN). Median and mean RB% values were 353.7 and 882.6, respectively.

**Figure 6:**
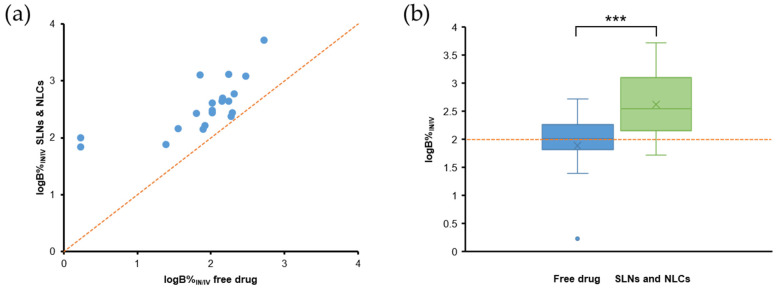
(**a**) Summary of paired logB%_IN/IV_ values for free drug (IN) and SLN and NLC-based formulation (IN) in the same studies (*n* = 20). The dot-line is for y = x. (**b**) Comparison of logB%_IN/IV_ between free drug (IN) (*n* = 20) and SLN and NLC-based formulation (IN) (*n* = 28) using boxplots. × indicates mean value. The horizontal dot-line is for logB%_IN/IV_ = 2. *** *p* < 0.001 (*t*-test).

In addition, we found that logB%_IN/IV_ values of SLN and NLC-based formulations (IN) were always higher than those of free drugs (IN) in same studies (Figure 6a). Mean logB%_IN/IV_ value of SLN and NLC-based formulations (IN) was 2.62, which was significantly higher than that (1.89) of free drugs (IN) (Figure 6b).

**Figure 7:**
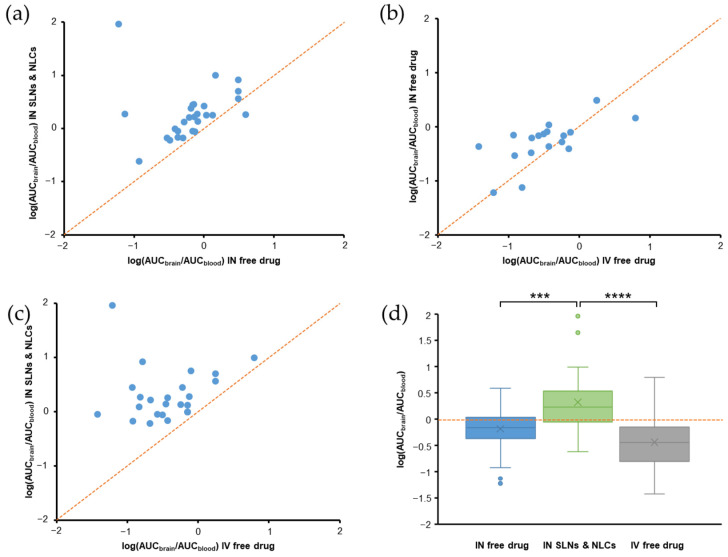
(**a**–**c**) Summary of paired AUC_brain_/AUC_blood_ ratios for free drug (IN), SLN and NLC-based formulation (IN), and free drug (IN) in the same studies. The dot-line is for y = x. *n* = 27 for (**a**), *n* = 19 for (**b**), and *n* = 24 for (**c**). (**d**) Comparison of AUC_brain_/AUC_blood_ ratios among free drug (IN) (*n* = 27), SLN and NLC-based formulation (IN) (*n* = 36), and free drug (IV) (*n* = 24) using boxplots. × indicates mean value. The horizontal dot-line is for logAUC_brain_/AUC_blood_ = 0 (i.e, AUC_brain_/AUC_blood_ = 1). *** *p* < 0.001 and **** *p* < 0.0001 (ANOVA with a Tukey’s HSD post-hoc test).

AUC_brain_/AUC_blood_ ratios of SLN and NLC-based formulation (IN) and free drugs (IN and IV) were compared. Regarding SLN and NLC-based formulations (IN) and free drugs (IN) (Figure 7a), only one of the 27 cases (streptomycin-loaded SLNs [72]) reported a higher AUC_brain_/AUC_blood_ ratio for the free drug (IN). Regarding free drugs (IN) and free drugs (IV) comparisons (Figure 7b), the IN route had a higher AUC_brain_/AUC_blood_ ratio in 14 of 19 cases. Five cases reported a higher AUC_brain_/AUC_blood_ ratio for free drugs (IV), which were piribedil-loaded SLN gel [27], efavirenz-loaded NLCs [65], ropinirole-loaded anionic NLC gel [61], nicergoline-loaded NLCs [58], and phenytoin-loaded NLCs (of particle size < 50 nm) [36]. The AUC_brain_/AUC_blood_ ratios of SLN and NLC-based formulations (IN) were always higher than those of free drug (IV) in same studies (Figure 7c).

The boxplots in Figure 7d show that log(AUC_brain_/AUC_blood_) values generally followed the order: SLN and NLC-based formulations (IN) > free drugs (IN) > free drugs (IV). The mean log(AUC_brain_/AUC_blood_) values of these systems were 0.32, −0.18, and −0.44, respectively, which were significantly different (*p* = 1.7 × 10^−7^, ANOVA). From post-hoc test results, we observed significant differences between SLN and NLC-based formulations (IN) and free drugs (IN) (*p* < 0.001) and between SLN and NLC-based formulations (IN) and free drugs (IV) (*p* < 0.0001), but the difference between mean log(AUC_brain_/AUC_blood_) values of free drugs (IN) and free drugs (IV) was not statistically significant (*p* > 0.05). Thus, IN administration increased AUC_brain_/AUC_blood_ ratios as compared with the IV route, and the incorporation of drugs in SLNs and NLCs further increased these ratios. Two SLN and NLC-based formulations (IN) reported exceptionally high AUC_brain_/AUC_blood_ ratios, which were phenytoin-loaded NLCs of different particle sizes (<50 nm and >100 nm) [36]. The high AUC_brain_/AUC_blood_ ratios observed in these cases was due to relatively low AUC_blood_ values. We speculate that the developed NLC systems sufficiently transported phenytoin to the brain via direct routes (DTP% > 99.8%), and thus, only a small amount of the drug entered the bloodstream.

## 7. Effects of Gelling Systems and Surface Modifications of SLNs and NLCs

### 7.1. Effects of Gelling Systems

Gelling systems can enhance drug nasal residence times and reduce enzymatic degradation and mucociliary clearance, and thus, improve nasal absorption and brain bioavailability of drugs [62]. In addition, mucoadhesive agents in gels can open tight junctions between epithelial cells and thereby enhance drug delivery to the brain [26]. Gelling systems are prepared using mucoadhesive polymers (e.g., HPMC or chitosan) and viscosity enhancers, which reduce mucociliary clearance and increase drug retention times in the nasal cavity [142]. Various biocompatible polymers have been used to prepare gelling systems for the IN route, such as Poloxamer 407, 188, methylcellulose, and HPMC [62]. These gelling systems can attenuate the nasal mucosal toxicities of nanosystems [143]. In the case of the cationic ropinirole-loaded NLCs, nasal mucosa toxicity disappeared after NLCs were loaded in Poloxamer thermosensitive in situ gels [61]. In most studies, SLNs and NLCs have been reported to be non-toxic to nasal mucosa, and thus, gelling systems would appear to primarily increase nasal residence times and minimize mucociliary clearance.

Gelling systems were used to improve the brain targeting efficiencies of SLNs and NLCs in 21 of the 81 studies. Most of these studies compared SLN and NLC gels (IN) with the free drug (IN and IV). In some studies, SLN and NLC gels (IN) were compared with SLN and NLC suspensions (IV) [26,74], which did not enable proper comparison of the efficacies of gelling systems over plain SLNs and NLCs.

Several studies compared SLN and NLC gels (IN) and plain SLN and NLC suspensions (IN). A recent study compared a piribedil-loaded SLN suspension and piribedil-loaded SLN gel, and found the brain bioavailability (AUC_0–6h,brain_) and mean brain residence time (MRT_brain_) of the SLN gel were 1.29- and 1.40-fold greater than those of the SLN suspension, respectively [27]. Furthermore, the DTE%, DTP%, logRDTE%, B%_IN/IV_, and RB% values of the SLN gel (IN) were 137.5, 27.29, 2.40, 145.3, and 404.5, higher than those of the SLN suspension (IN) (119.9, 16.59, 2.34, 112.3, and 312.6, respectively). These findings indicate that the SLN gel (IN) better targeted brain than the SLN suspension (IN). Besides the longer nasal residence of gel (21.7 min versus 8 min), the use of thermoresponsive in situ gel with rapid gelation may have reduced the spread of formulation from the olfactory region to respiratory epithelium, and therefore increased drug transport via direct routes [27]. In another study, the occurrence of seizures in pentylenetetrazole-treated rats was lower for lorazepam-loaded NLC gel (IN) than a lorazepam-loaded NLC suspension (IN) [84].

However, gelling systems are not always effective for SLNs and NLCs. In a previous study, agomelatine-loaded SLNs (IN) were found to have DTE%, DTP%, and B%_IN/IV_ values of 190.02, 47.379, and 83.26, respectively [67], and when incorporated into an in situ gel of Poloxamer 407 and 0.4% HPMC, these values reduced to 141.42, 29.29, and 52.6, respectively. A recent study reported that cannabidiol-loaded NLCs (IN) increased antinociceptive effects in mice with neuropathic pain [139]. However, NLC gel had no antinociceptive effect because the hydrophilic gel reduced the diffusion and nasal absorption of the lipophilic cannabidiol.

### 7.2. Effects of Surface Modification of SLNs and NLCs

Nasal formulations generally exploit mucoadhesive excipients to minimize mucociliary clearance and increase residence times of drugs [144]. These excipients are usually hydrophilic polymers, such as gums, alginates, starch, gelatin, chitosan, chitosan derivatives and conjugates, sodium hyaluronate, methylcellulose, HPMC, carboxymethylcellulose, polyacrylates, polymethacrylates, and crospovidone [145,146,147,148,149], which are able to interact with mucus by hydrophobic interactions, electrostatic attraction, hydrogen, and van der Waals bonds [150]. For SLNs and NLCs, these polymers are used to coat the surfaces of nanoparticles. Twelve of the 81 studies used a surface modification to improve the brain targeting efficiencies of SLNs and NLCs (IN). In 75% of these studies, chitosan or its derivatives (glycol chitosan, TMC) were used, which can cause a change from negative to positive surface charge of SLNs and NLCs and increase electrostatic attractions between nanoparticles and mucus [151]. In addition, these excipients can induce opening of tight junctions between nasal epithelial cells to enhance drug transport [152].

Most of the 12 studies compared coated SLNs and NLCs (IN) with free drugs (IN and IV). Several studies clarified the efficacies of surface modification by comparing coated SLNs and NLCs (IN) with uncoated SLNs and NLCs (IN). A previous study reported that the mucoadhesive strength (as determined by detachment force) of ferulic acid-loaded SLNs increased from 6.88 to 8.55 N after coating with chitosan [90]. In addition, the drug concentration in the brain of CS-SLNs (IN) was 1.27-fold higher than that of SLNs (IN), suggesting the efficacy of chitosan surface modification in nose-to-brain delivery. Likewise, CS-SLNs (IN) improved cognitive ability and biochemical levels in the cortex and hippocampus better than the SLNs (IN) in the Alzheimer’s disease-induced rats. In a previous study, asenapine-loaded NLCs were coated with glycol CS [29], and in rat PK studies, the C_max,brain_ of GC-NLCs (IN) was 1.3-fold higher than that of NLCs (IN). In addition, DTE%, DTP%, logRDTE%, logRDTP%, B%_IN/IV_, and RB% values of GC-NLCs (IN) were higher than those for NLCs (IN) [28], which indicated that the glycol CS coating effectively increased brain targeting of NLCs. A recent study reported the efficacy of NLCs and TMC-NLCs co-loaded with artemether and lumefantrine [64]. It was found that TMC-NLCs (IN) exhibited greater parasite suppression than NLCs (IN) (95% vs. 82.5%) in parasite-injected mice.

## 8. Challenges, Considerations, and Future Developments

SLN and NLC-based formulations have been developed and evaluated for nose-to-brain delivery in various studies, because they increase nasal retention, reduce mucociliary clearance, improve drug solubility and permeability, minimize drug enzymatic degradation, and enhance nasomucosal biocompatibility [57,58,59,60]. Accumulating data demonstrates that SLNs and NLCs are effective drug delivery systems that can transport drugs to the brain via direct routes. However, some challenges need to be overcome since successful SLN and NLC-based formulations at the preclinical stage may fail to perform at the clinical stage for many reasons. First, the nasal cavities of humans and animal models differ anatomically. Nasal cavity length, surface areas volumes histology, and geometry are species-dependent and affect the retention and absorption of drugs [7,40]. The majority of studies included in this review used rats and mice in PK and PD studies due to their low cost and accessibility. However, their nasal cavities are very different from those of humans and other animals, such as rabbits, sheep, monkeys, and dogs. Rats and mice have small nasal orifices, which cause difficulties during intranasal administration, while those of rabbits, sheep, monkeys, and dogs are considerably larger [153]. The olfactory region comprises ~10% of the nasal cavity in humans, rabbits, sheep, and monkeys, whereas, in mice, rats, and dogs, it occupies up to 50% [33]. Second, volumes of IN administrations are also species dependent and vary from ~10 μL for mice, 40–50 μL for rats, and greater amounts for other larger animals. Furthermore, the tools used for administration include micropipettes, syringes, nasal atomizers, sprays, and cannulas, which may affect the overall drug absorption and therapeutic effects [154]. Third, the experimental procedure for PK studies on nose-to-brain delivery via IN administration vary among research groups, and different approaches have been used to investigate the brain targeting efficiencies of developed formulations. Therefore, the methods used for PK studies on formulations intended for nose-to-brain delivery be standardized to some extent.

To develop SLN and NLC-based formulations for nose-to-brain delivery, some matters should be taken into consideration. In vivo PK studies on SLN and NLC-based formulations can be carried out on mice and rats during the initial stages, but rabbits, sheep, monkeys, or dogs should be used during later stages [154]. PK studies need to be performed using SLN and NLC-based formulations (IN) and free drugs (IN and IV) to determine relevant parameters such as DTE%, DTP%, B%_IN/IV_, and RB%. Some developed SLN and NLC-based formulations have B%_IN/IV_ values of <100 despite favorable DTE% and DTP% values [60,61,67,112]. Therefore, a formulation is deemed to have potential if it meets all required parameters. Furthermore, considerations of PK and PD data are critical in terms of predicting the feasibility of SLN and NLC-based formulations. The incorporation of gelling systems or surface modifications is favorable since they can improve drug nasal absorption and brain bioavailability. However, they are not always effective for SLNs and NLCs [67,139], and thus, investigations and comparisons of plain SLNs or NLCs and coated systems or gels are necessary. Another consideration is that the tools and techniques used for administration can affect drug absorption and therapeutic effects. Formulations should be administered to the posterior-most region of the nasal cavity (the olfactory region) to enable access to the olfactory pathway and minimize mucociliary clearance [142]. Regular nasal drops can increase drug exposure in the respiratory region, which results in more drugs entering the systemic circulation. Some delivery devices, such as syringeless needles and sprays, can deliver drug to the deeper, posterior region. Therefore, SLN and NLC-based formulations should be developed using appropriate delivery devices. Finally, an upward, tilted head position can maximize olfactory region exposure and enhance drug absorption [62]. Some transporters (e.g., organic anion transporter 3 and organic cation transporter 2) may contribute to the drug delivery from nose to brain and need to be considered during formulation development [155].

SLNs and NLCs for nose-to-brain drug delivery have been increasingly developed during recent years. The results summarized from 81 in vivo PK and PD studies demonstrate the efficacy of SLN and NLC-based formulations for nose-to-brain delivery and their potentials in clinical use. The transition from preclinical studies in animals to clinical studies is a great challenge that requires precise and accurate methods. Currently, some nasal formulations are in clinical trials or have been approved for medical use [33]. However, to the best of our knowledge, none of them are SLN and NLC-based formulations. Although SLN and NLC-based formulations have not been subjected to clinical studies, preclinical studies to date have demonstrated the undeniable potentials of these formulations. We believe that SLN and NLC-based formulations will be subjected to clinical trials soon and that they will enhance the management of different CNS diseases in the near future.

## Figures and Tables

**Table 1:** Major features of SLN and NLC-based formulations for nose-to-brain delivery: PK studies with DTE% and DTP%.

Drug	Formulation	Animal	DTE%	DTP%	logRDTE%	logRDTP%	B%_IN/IV_	RB%	Other Outcomes	Ref.
Almotriptan	SLNs (Precirol, PVA), gel of 18% Plx 407 + 0.75% Na-CMC	Rats	335.7	70.21	**2.12**	**2.06**	**237.0**	**125.9**	Fast onset (T_max,brain_ = 10 min); safety (biomarkers’ evaluation and histopathological examination)	[99]
Agomelatine	SLNs (Gelucire 43/01, PVA, sodium deoxycholate)	Rats	190.0	47.37	ND	ND	**83.3**	ND	Higher systemic bioavailability (2.76-fold vs. oral susp.)	[67]
	SLNs in gel of 16% Plx 407 + 0.4% HPMC	Rats	141.4	29.29	ND	ND	**52.6**	ND	Higher systemic bioavailability (2.35-fold vs. oral susp.); prolonged half-life (plasma, 378.92 min)	[60]
Artemether	NLCs (Trimyristin, medium chain triglyceride, Plx 188)	Rats	278.2	64.02	**2.07**	**2.05**	**444.8**	**254.5**	Higher brain:blood conc. ratio (vs. IN and IV drug sol.); safety (histopathological examination)	[108]
Asenapine	NLCs (GMS, oleic acid, Tween 80)	Rats	207.2	**51.73**	**2.07**	**2.08**	**276.7**	**267.8**	Higher C_max,brain_ (1.4- and 1.8-fold vs. IN and IV drug sol., respectively)	[28]
Asenapine	NLCs (GMS, oleic acid, Tween 80), glycol CS coating	Rats	288.3	**65.31**	**2.22**	**2.18**	**407.9**	**394.8**	Higher C_max,brain_ (1.8- and 2.3-fold vs. IN and IV drug sol., respectively); safety (histopathological examination and embryo fetal toxicity study)	[29]
Donepezil	SLNs (Glyceryl behenate, Tween 80, Plx 188)	Rats	288.8	65.37	**2.26**	**2.26**	**163.6**	197.6	Higher C_max,brain_ (4.1-fold vs. IN drug sol. and 5.4-fold vs. IV drug sol.)	[109]
Donepezil	SLNs (GMS, Tween 80, Plx 188)	Rabbits, rats	553.9	81.94	**2.36**	**2.14**	**300.9**	**290.9**	Higher C_max,brain_ (5.5-fold vs. IN drug sol. and 7.6-fold vs. IV drug sol.); higher drug in brain vs. IN drug sol. (gamma scintigraphy)	[110]
Duloxetine	NLCs (GMS, capryol PGMC, Plx 188, sodium taurocholate)	Rabbits, rats	758.1	86.81	**2.42**	**2.12**	**5219**	**984.9**	Higher drug conc. in brain (3.8- and 2.9-fold vs. IN drug sol.)	[73,92,102]
Efavirenz	NLCs (Precirol ATO 5, Captex P500, MYS-25)	Rats	**1205** (487.4)	**91.7** (88.2)	**3.40**	ND	**1272.5**	1782.5	Higher C_max,brain_ (2.5-fold vs. IN drug susp.); higher AUC_0-inf,brain_ (3.73-fold vs. IV NLCs); safety (biomarkers evaluation and histopathological examination)	[65]
Escitalopram + paroxetine	NLCs (Precirol ATO5, Lauroglycol 90, Tween 80, borneol), gel of 18% Plx 407, 0.2% Carbopol 974P	Mice	25.4; 388	−293.7; 74.23	ND; **2.22**	ND; **2.12**	ND; **272.5**	ND; **138.3**	Higher C_max,brain_ (4.8-fold vs. IN free drug gel and 5.9-fold vs. IV drug sol.) for paroxetine	[111]
Haloperidol	SLNs (GMS, Tween 80)	Rats	2362.4	95.77	**2.32**	**2.02**	**500.9**	349.7	Higher C_max,brain_ (3.7-fold vs. IN drug sol. and 4.3-fold vs. IV drug sol.)	[103,104]
Levofloxacin + doxycycline	SLNs (Compritol 888 ATO, stearic acid, span 60, Plx 407), gel of 1% HPMC	Rats	149.8; 161.9	33.28; **38.26** (40.24)	**2.19;** **2.21**	ND	**76.4;** ND	**311.0;** ND	Lower C_max,brain_ and AUC_0–360min,brain_ of both drugs (vs. IV drug sol.)	[112]
Nicergoline	NLCs (Precirol ATO5, sesame oil, Tween 80)	Rats	**237.3** (187.3)	**57.87** (56.64)	**2.41**	ND	**266.3**	**417.6**	Higher AUC_0–8h,brain_ (1.44-fold vs. IV NLCs)	[58]
Ondansetron	NLCs (GMS, Capryol 90, soya lecithin, Plx 188), coating with *Delonix regia* gum	Rats	**5062** (506)	**98.02** (97.14)	ND	ND	**3432**	ND	Higher C_max,brain_ (4.1-fold vs. IV drug sol.); safety (histopathological examination)	[113]
Phenytoin	NLCs (Cholesterol, oleic acid, Plx 188)	Rats	**149,952**	**99.93**	**5.18**	ND	**4873**	**3025**	Safety (histopathological examination)	[36]
		Rats	**72,615**	**99.86**	**4.87**	ND	**3629**	**2252**		
Piribedil	SLNs (Palmitic acid, polyvinyl alcohol)	Rats	119.9	16.59	**2.34**	ND	**112.3**	**312.6**	Reduced C_max,plasma_ vs. IN drug susp.	[27]
	SLNs in gel of 1.5% methyl cellulose		137.5	27.29	**2.40**	ND	**145.3**	**404.5**		
Risperidone	SLNs (Compritol 888 ATO, Plx 407)	Mice	**830.9**	**87.97**	ND	ND	**2278.6**	ND	Higher brain:blood ratio at 1 h (10-fold vs. IV drug sol. and 5-fold vs. IV SLNs); brain targeting confirmed by gamma scintigraphy images	[66]
Risperidone	NLCs (Stearic acid, oleic acid, Tween 80), CS coating	Rats	**252.7**	**60.4**	**2.37**	**2.98**	**440.3**	**308.9**	Higher drug permeation ex vivo (2.32-fold vs. drug susp.)	[63]
Ropinirole	Anionic NLCs (Compritol 888 ATO, Labrafac, PC, Plx 188, Tween 80, SDS), gel of 15% Plx 407, 12% Plx 188, 1% HPMC	Rats	158.5	36.9	**2.83**	ND	**69.8**	4087.9	Higher C_max,brain_ (48- and 81.8-fold) and half-life (5- and 8.8-fold) for cationic and anionic NLC gels vs. IN drug sol., respectively; safety (histopathological examination)	[61]
	Cationic NLCs (+stearic acid), gel of 15% Plx 407, 12% Plx 188, 1% HPMC	Rats	128.6	22.3	**2.74**	ND	**99.4**	5820.3		
Sesamol	SLNs (GMS, Tween 80)	Rats	764	86.1	ND	ND	**590.4**	ND	Shorter T_max,brain_ (10 min vs. 30 min for IV drug sol.); higher C_max,brain_ (13.2-fold vs. IV drug sol.)	[114]
Sumatriptan	NLCs (Stearic acid, cholesterol, triolein, Brij 35, Brij 72)	Rats	**2416** (258.0)	**95.86** (61.23)	**2.60**	**2.06**	**1295**	**744.6**	Higher C_max,brain_ (5.6-, 7.3-, and 9.4-fold vs. IN drug sol., IV drug sol., and IV NLCs, respectively); higher AUC_0–4h,brain_ (7.70-fold vs. IV NLCs)	[115]
Tarenflurbil	SLNs (GMS, stearic acid, soya lecithin, Tween 20)	Rats	183.2	45.4	**2.20**	**2.55**	**142.3**	**182.1**	Higher C_max,brain_ (1.5-, 1.7-, and 4.1-fold vs. IV drug sol., IN drug sol., and oral drug susp., respectively); higher drug level in brain (multiple-dose, ~2-fold vs. IV drug sol. and oral drug susp.)	[68]
Temozolomide	NLCs (Gelucire 44/14, α tocopherol, Tween 80)	Rats	457.8	**78.16**	**2.61**	**2.82**	**588.1**	**282.7**	Gamma scintigraphy images confirmed the brain accumulation of NLCs	[116]
Tenofovir	NLCs (Compretol 888 ATO, oleic acid, Tween 80, Plx 188)	Rats	**481.9**	**79.25**	**2.23**	**2.08**	**1204**	**402.7**	Higher C_max,brain_ (3.2, 5.8- and 6.5-fold vs. IV NLCs, IV drug sol., and IN drug sol., respectively); higher AUC_0-inf,brain_ (3.6-fold vs. IV NLCs); higher brain accumulation (confocal microscopic image, vs. IV NLCs); safety (histopathological examination)	[78]
Ziprasidone	NLCs (Gelucire 43/01, Capmul MCM, Labrasol, Transcutol P)	Rats	476.8	**79.0** (89.85)	ND	ND	**ND**	ND	Higher brain:blood conc. ratios and faster onset (10 min) vs. IV drug sol.	[117]

PS, mean particle size; PI, polydispersity index; EE, entrapment efficiency; ZP, zeta potential; sol., solution; susp., suspension; conc., concentration; ND: not determined; PVA, polyvinyl alcohol; Plx, Poloxamer; Na-CMC: Sodium carboxymethyl cellulose; HPMC, hydroxypropyl methylcellulose; PC, phosphatidylcholine; SDS, deoxycholic acid sodium salt; GMS, glycerol monostearate; CS: chitosan. Bold values for DTE%, DTP%, logRDTE%, logRDTP%, B%_IN/IV_, and RB% indicate that the values were calculated or recalculated in this review using raw data from the original studies. In the cases of recalculation, both reported and recalculated values are presented.

**Table 2:** Major features of SLN and NLC-based formulations for nose-to-brain delivery: Comparison using brain bioavailability from PK studies (without DTE% and DTP%).

Drug	Formulation	Animal	Outcomes	Ref.
Almotriptan	NLCs (Compritol, Labrafil, Tween 80, Lauroglycol), CS coating	Rabbits	Higher C_max,brain_ (7.6-fold) and AUC_0–8h,brain_ (8.1-fold) vs. IN drug sol.; fast onset (T_max,brain_ = 10 min); higher brain:blood conc. ratios (vs. IN drug sol.); safety (histopathological examination)	[70]
Alprazolam	SLNs (GMS, Tween 80, Plx 188)	Rabbits, rats	Higher AUC_0–8h,brain_ (1.33-fold vs. IV SLNs and 1.99-fold vs. IN drug sol.); reduced drug accumulation in liver, spleen, intestine, and kidney (vs. IV SLNs)	[38]
Astaxanthin	NLCs (GMS, soybean oil, Plx 188), gel of 20% Plx 407 + 0.5% CS	Rats	Higher C_max,brain_ (9.5-fold) and AUC_0–24,brain_ (7.8-fold) vs. IN free drug gel	[97]
Buspirone	SLNs (Compritol 888 ATO, Tween 80, Plx 188)	Rats	Higher C_max,brain_ (1.7-fold vs. IV drug sol. and 2.3-fold vs. IV SLNs); higher AUC_0–24h,brain_ (2.2-fold vs. IN drug sol.)	[77]
Buspirone	NLCs (GMS, oleic acid, Tween 80), CS coating	Rats	Higher C_max,brain_ (1.5-fold vs. IV drug sol. and 2.6-fold vs. IV NLCs); higher AUC_0–12h,brain_ (2.2-fold vs. IV drug sol. and 3.1-fold vs. IV NLCs)	[118]
Cinnarizine	NLCs (Cetyl palmitate, oleic acid, 3% Plx 188 + soya lecithin), gel of 19% Plx 407 + 0.5% Plx 188 + 0.1% CS	Rats	Higher C_max,brain_ (2.07-fold) and AUC_0–4h,brain_ (2.23-fold) vs. IN drug sol.	[119]
Clozapine	NLCs (Precirol ATO 5, oleic acid, Tween 80)	Mice	Higher C_max,brain_ (11.8-fold) and AUC_0–12h,brain_ (6.15-fold) vs. oral clozapine tablet; safety (histopathological examination)	[69]
Curcumin	NLCs (Precirol ATO 5, Capmul MCM, Tween 80, soya lecithin)	Rats	Higher C_max,brain_ (1.6-fold) and AUC_0–48h,brain_ (2.2-fold) vs. IN drug susp.; safety (histopathological examination)	[120]
Donepezil	NLCs (Glyceryl distearate, Capmul MCM, Acrysol K150, Plx 188, Tween 80), gel of gellan gum	Rats	Higher AUC_0–8h,brain_ (126-fold vs. oral tablet)	[87]
Flibanserin	NLCs (Compritol 888 ATO, sweet almond oil, PC, Gelucire 44/14), gel of 0.6% gellan gum	Rats	Higher C_max,brain_ (3.5-fold) and AUC_0-inf,brain_ (6.3-fold) vs. IN flibanserin gel; safety (histopathological examination)	[121]
Lurasidone	NLCs (Gelot 64, Capryol 90, Tween 80, Transcutol P)	Rats	Higher C_max,brain_ (1.9- and 7.9-fold) and AUC_0–24h,brain_ (2.96- and 9.3-fold) vs. IN drug sol. (IN) and oral drug susp., respectively.	[122]
Olanzapine	NLCs (Compritol 888 ATO, Labrafil M 1944 CS, Tween 80), gel of 17% Plx 407 + 0.3% HPMC	Mice	Higher C_max,brain_ (3.98-fold) and AUC_0–6h,brain_ (3.81-fold) vs. IV drug sol.; safety (hematological study and histopathological examination)	[26]
		Rats	Higher AUC_0-inf,brain_ vs. IN microemulsion gel and IV NLCs; safety (hematological study and histopathological examination)	[75]
Oleuropein	NLCs (Tefose, Capmul, Plx 188, Tween 80, soy lecithin)	Rats	Higher AUC_0–6h,brain_ (2.23-fold vs. IV NLCs); safety (histopathological examination)	[123]
Pueraria flavones	SLNs (Borneol-stearic acid, lipoid E80, Plx 188)	Rats	Higher AUC_0–8h,brain_ (8.31-fold) and C_max,brain_ (8.29-fold) (IN borneol-stearic acid SLNs vs. IN SLNs)	[124]
Quetiapine	NLCs (Gelucire 44/14, oleic acid, Tween 80, Transcutol P)	Rats	Higher C_max,brain_ (4.15-fold) and AUC_0–6h,brain_ (3.57-fold) vs. IV NLCs; safety (histopathological examination)	[125]
Resveratrol	NLCs (Cetyl palmitate, Capmul MCM, Acrysol, Tween 80, Plx 188), gel of gellan and xanthan gum	Rats	Higher C_max,brain_ (2.6-fold) and AUC_0–8h,brain_ (1.4-fold) vs. oral drug susp.; safety (histopathological examination)	[85,86]
Rizatriptan	SLNs (Glycerol monostearate, lecithin, Plx 407)	Rats	Higher C_max,cerebrospinal fluid_ (1.3- and 5.5-fold) and AUC_0-inf,cerebrospinal fluid_ (1.7- and 3.0-fold) vs. IV drug sol. and oral tablet, respectively	[126]
Ropinirole	NLCs (Tristearin, flaxseed oil, TPGS, Lipoid S100), TMC coating	Mice	Higher C_max,brain_ (1.7-fold vs. IV NLCs and 17.4-fold vs. IN drug sol.); higher AUC_0–12h,brain_ (1.5-fold vs. IV NLCs and 13.7-fold vs. IN drug sol.); safety (histopathological examination)	[127]
Streptomycin	SLNs (Compritol 888 ATO, Tween 80, soy lecithin)	Rabbits; mice	Higher brain conc. (4.57-fold at 0.5 h and 6.0-fold at 24 h) and AUC_0-inf,brain_ (3.6-fold) vs. drug sol. in mice; higher brain conc. in rabbits (gamma scintigraphy)	[72]
Teriflunomide	NLCs (Glyceryl dibehenate, glyceryl mono-linoleate, Gelucire 44/14), gel of 0.2% carbopol 974P + 0.2% gellan gum	Mice	Higher AUC_0–8,brain_ (1.34-fold vs. IV NLCs); higher brain:blood conc. ratios (2–3- and 8–10-fold vs. IN and IV NLCs, respectively); safety (histopathological examination and biochemical markers)	[74]

TPGS, D-α-Tocopherol polyethylene glycol 1000 succinate; TMC, N,N,N-trimethyl chitosan.

**Table 3:** Major features of SLN and NLC-based formulations for nose-to-brain delivery: Comparison using brain:blood concentration ratios from PK studies.

Drug	Formulation	Animal	Outcomes	Ref.
Efavirenz	SLNs (Tripalmitin, Plx 188)	Rats	Higher brain:blood conc. ratio at 24 h (150-fold vs. oral tablet)	[128]
Pioglitazone	NLCs (Tripalmitin, Capmul MCM, stearyl amine, Tween 80, Plx 188)	Rats	Higher brain:blood conc. ratio (1.9- and 10.7-fold vs. IN and IV drug sol., respectively); safety (histopathological examination);	[129]
Rimonabant	NLCs (Tristearin, Miglyol 812N, Plx 188)	Rats	Higher brain:blood conc. ratio (vs. IN drug sol.)	[130]
Valproic acid	NLCs (Cetyl palmitate, soy lecithin, octyldodecanol, Plx 188)	Rats	Higher brain:plasma conc. ratio at 60 min (5.09-fold vs. IP NLCs)	[80]

IP, intraperitoneal.

**Table 4:** Major features of SLN and NLC-based formulations for nose-to-brain delivery: Comparison using drug accumulation in the brain from PK studies.

Drug	Formulation	Animal	Outcomes	Ref.
Artemether + lumefantrine	NLCs (Gelucire 50/13, Lipoid S75, oleic acid, Capmul MCM, Tween 80), TMC coating	Mice	Higher drug conc. in mice brain (vs. IN and oral drug susp.)	[64]
Astaxanthin	SLNs (Stearic acid, Plx 188, lecithin)	Rats	Higher drug conc. in the brain (~2-fold vs. IV SLNs)	[71]
Dimethyl fumarate	SLNs (Tristearin, Tween 80, Plx 188)	Mice	Similar brain accumulation to SLNs (IP) at a 10-fold lower dose	[131]
Embelin	NLCs (Cetyl palmitate, octyldodecanol, Plx 188)	Rats	Higher drug conc. in the brain (vs. IN drug sol. and IV marketed formulation)	[82]
Ferulic acid	SLNs (Compritol, Plx 188), CS coating	Rats	Higher drug conc. in brain (6.91-fold for IN CS-SLNs and 5.42-fold for IN SLNs vs. IN drug susp.); safety (histopathological examination)	[90]
Lamotrigine	NLCs (GMS, oleic acid, Tween 80, Plx 188)	Rats	Higher drug conc. in the brain (1.4- and 5.1-fold vs. IN and oral drug sol.)	[81]
Naloxone	SLNs (GMS, Plx 407, Tween 80)	Rabbits, rats	Better brain deposition via gamma scintigraphy and biodistribution studies (vs. IN drug sol.); safety (weight variation, histopathological examination)	[132]
Paeonol	SLNs (GMS, soybean lecithin, Plx 407, Tween 80), gel of 0.4% deacetylated gellan gum + 0.3% HPMC	Rats	Higher brain accumulation (vs. IV SLNs)	[12]
Quetiapine	SLNs (GMS, span-80, butanol), gel of 21% Plx 407 + 5.6% Plx 188	Rats	Drug conc. in the brain: similar to those for IV drug sol. and higher than those for oral drug sol.; better effect in improving hippocampal morphology change	[133]
Rivastigmine	NLCs (GMS, Capmul MCM C8, Lecithin and Tween 80), gel of 15% Plx 407 + 0.8% gellan gum	Rats, mice	Higher drug concentration in the brain at 1 h (4.6-, 8.6-, and 1.6-fold vs. IN drug sol., IV drug sol., and IV NLCs, respectively); safety (hematology and histopathological examination)	[88]
Geraniol-ursodeoxycholic acid	SLNs (Compritol ATO 888, Span 85, Tween 80, taurocholate sodium salt)	Rats	Detection of drug in the cerebrospinal fluid until 3 h; safety (histopathological examination)	[134]
hIGF-1	NLCs (Precirol ATO5, Miglyol, Tween 80, Plx 188), CS coating	Mice	Brain accumulation (via fluorescence imaging); safety (histopathological examination)	[76]
Nalbuphine	SLNs (Phosphatidylcholine, Tween 80, Plx 407)	Rats	Detection of drug in brain from 10 min to 8 h	[135]
Ondansetron	SLNs (GMS, Plx 188, lecithin)	Rabbits	Rapid drug localization in brain (1 h, gamma scintigraphy); safety (histopathological examination)	[136]
Rosmarinic acid	SLNs (GMS, Tween 80, hydrogenated soya phosphatidyl choline)	Rats	Drug amount in brain (5.69 µg)	[137]
Zolmitriptan	SLNs (Steari acid, cholesterol, lecithin), gel of 3% HPMC	Rats	Accumulation of SLNs in brain until 24 h	[138]

IM, intramuscular; hIGF-1, human insulin-like growth factor-1.

**Table 5:** Major features of SLN and NLC-based formulations for nose-to-brain delivery: PD studies.

Drug	Formulation	Animal Model	Outcomes	Ref.
Artemether + lumefantrine	NLCs (Gelucire 50/13, Lipoid S75, oleic acid, Capmul MCM, Tween 80), TMC coating	Plasmodium berghei ANKA-injected mice	Higher parasite suppression (95% vs. 82.5% (IN NLCs), 79.1% (IN drug susp.), and 46.3% (oral drug susp.))	[64] *
Artesunate	NLCs (Compritol HD5 ATO, Phospholipon 90H, Miglyol 812 N, Transcutol HP, Tween 80, Plx 188)	Plasmodium berghei ANKA-injected mice	Similar activity (54.70% vs. 58.80%) and reduction in parasitaemia (33.28% vs. 42.18%) vs. IM NLCs	[57]
Asenapine	NLCs (GMS, oleic acid, Tween 80)	Rats with L-dopa and carbidopa-induced catalepsy	Better therapeutic and safety profiles (vs. IN drug sol.)	[28] *
Astaxanthin	NLCs (GMS, soybean oil, Plx 188), gel of 20% Plx 407 + 0.5% CS	Rats with haloperidol-induced catalepsy	Improved behaviors in rotarod test and akinesia measurement (vs. IN free drug gel)	[97] *
Cannabidiol	NLCs (Stearic acid, oleic acid, Span 20, cetylpyridinium chloride), gel of 17% Plx 407 + 3% Plx 188	Mice with paclitaxel-induced neuropathic pain	Increased antinociceptive effects (vs. IN and oral drug sol.)	[139]
Carbamazepine	NLCs (Precirol, Capmul MCM, Tween 80, Span 20), gel of 20% Plx 407, 5% Plx 188, 0.2% CS	Rats with MES	Higher protection efficacy against seizure (vs. IN plain drug gel)	[79]
Cinnarizine	NLCs (Cetyl palmitate, oleic acid, 3% Plx 188 + soya lecithin), gel of 19% Plx 407 + 0.5% Plx 188 + 0.1% CS	Formalin-induced acute nociception rats	Higher antinociceptive activity in neurogenic pain and inflammatory pain (vs. IN drug sol.)	[119] *
Clonazepam	SLNs (GMS, stearic acid, compritol, oleic acid, glycerol oleate), gel of 15% Plx 407 + 0.75% sodium alginate	Mice with pentylenetetrazole-induced epilepsy	Prolonged onset times for convulsion (7.5- and 1.5-fold) and death (14- and 5-fold) for IN SPION-NLC gel and IN NLC gel vs. control	[83]
Donepezil	NLCs (Glyceryl distearate, Capmul MCM, Acrysol K150, Plx 188, Tween 80), gel of gellan gum	Rats with scopolamine-induced amnesia	Improved cognitive function (vs. oral tablet)	[87] *
Duloxetine	NLCs (GMS, capryol PGMC, Plx 188, sodium taurocholate)	Rats, locomotor activity and forced swimming tests	Improved locomotor activity, increased swimming and climbing time, reduced immobility period vs. drug sol. (IN and IV);	[73,92,102] *
Embelin	NLCs (Cetyl palmitate, octyldodecanol, Plx 188)	Rats with pentylenetetrazole-induced epilepsy	Reduced malondialdehyde and nitrite and increased glutathione (vs. IN drug sol. and IV marketed formulation)	[82] *
Ferulic acid	SLNs (Compritol, Plx 188), CS coating	Rats with streptozocin-induced Alzheimer’s disease	Improved cognitive ability and biochemical parameters (IN CS-SLNs > IN SLNs > IN, oral drug susp., oral SLNs)	[90] *
Fluoxetine	NLCs (Precirol ATO5, Lauroglycol 90, Tween 80)	Mice, marble-burying and forced swimming tests	Reduced depressive and anxiety-like behaviors (better than oral drug solution)	[93]
GDNF	NLCs (Precirol ATO5, Miglyol, Tween 80, Plx 188), CS coating	6-hydroxydopamine partially lesioned rats	Increased behavioral improvement, neuroprotective, and neuro-restorative effects (vs. oral drug sol.)	[95]
GDNF	NLCs (Precirol ATO5, Mygliol, Tween 80, Plx 188), coating with transactivator of transcription (TAT) peptide-CS conjugate	Mice with 1-methyl-4-phenyl-1,2,3,6-tetrahydropyridine-induced Parkinson’s disease	Better motor recovery and immunohistochemistry data (vs. IN GDNF sol.)	[94]
Ketoconazole	NLCs (Compritol 888 ATO, Miglyol 812 N, Solutol HS15, Tween 80)	Mice infected with fungal cells	Reduced fungal burden in brain (vs. IN drug sol.)	[140]
Lamotrigine	NLCs (GMS, oleic acid, Tween 80, Plx 188)	Rats with MES	Improved behavioral abnormalities, decreased malondialdehyde, and increased glutathione (vs. IN and oral drug sol.)	[81] *
Lorazepam	SLNs (GMS, oleic acid, Plx 407), gel of CS and β-glycerol phosphate	Rats with pentylenetetrazole-induced epilepsy	Reduced occurrence seizures (vs. IN NLC dispersion and IP drug sol.)	[84]
Nalbuphine	SLNs (Phosphatidylcholine, Tween 80, Plx 407)	Thermal allodynia induced rats	Better analgesic effect and early onset of action (vs. IM drug sol.)	[135] *
Resveratrol	NLCs (Cetyl palmitate, Capmul MCM, Acrysol, Tween 80, Plx 188), gel of gellan and xanthan gum	Rats scopolamine-induced amnesia	Improved memory function (vs. oral drug sol.)	[85,86] *
Risperidone	NLCs (Stearic acid, oleic acid, Tween 80), CS coating	Rats with haloperidol-induced catalepsy	Greater bio-efficacy (vs. IN and IV drug susp.)	[63] *
Rivastigmine	NLCs (Compritol 888 ATO, triacetin, sucrose acetate, Plx)	Rats with scopolamine-induced amnesia	Improve escape latency and transfer latency (vs. IN drug sol.)	[89]
Rivastigmine	NLCs (GMS, Capmul MCM C8, Lecithin and Tween 80), gel of 15% Plx 407 + 0.8% gellan gum	Mice with scopolamine-induced amnesia	Faster regain of memory loss (vs. IN and IV drug sol.)	[88] *
Ropinirole	SLNs (Dynasan 114, stearylamine, Plx 188, soy lecithin)	Mice with chlorpromazine-induced Parkinsonism-like signs	Better anti-tremor activity with a 3.3-fold lower dose (vs. oral tablet); safety (histopathological examination)	[96]
Rosmarinic acid	SLNs (GMS, Tween 80, hydrogenated soya phosphatidyl choline)	Rats with 3-nitropropionic acid-induced neurotoxicity	Increased protection against striatal oxidative stress (vs. IV SLNs)	[137] *
Selegiline	NLCs (Stearylamine, olive oil, Tween 80, Plx 188)	Rats with rotenone-induced Parkinson’s disease	Better restored behavior (vs. IN drug sol.); reduced malondialdehyde and nitrite and increased glutathione (vs. IN drug sol.)	[98]
Sertraline	SLNs (GMS, Plx 188, Tween 80)	Rats, tail suspension test and forced swimming test	Similar reduction in immobility duration (vs. IN free drug) but at a 2.5-fold higher dose.	[91]
Teriflunomide	NLCs (Compritol 888 ATO, maisine 35–1, Gelucire 44/14); gel of 17% Plx 407 + 0.3% HPMC	Rats with cuprizone-induced demyelination	Rapid remyelination and improved behaviors (vs. oral NLCs); safety (microscopic examination, hepatic biomarkers)	[100]
URB597	SLNs (Tristearin, Plx 188)	Rats, social behavioural study	Similar behavioral effects (vs. IP drug sol.)	[141]
Valproic acid	NLCs (Cetyl palmitate, soy lecithin, octyldodecanol, Plx 188)	Rats with MES	Similar protective effects (vs. IP drug sol.) with a 37.5-fold lower dose	[80] *

GDNF, glial cell-derived neurotrophic factor; MES, maximal electroshock-induced seizure; *, studies with PK studies that were presented in Table 1, Table 2, Table 3 and Table 4.

## Data Availability

The data presented in this study are available in this article.

## Supplementary Materials

The following supporting information can be downloaded at: https://www.mdpi.com/article/10.3390/pharmaceutics14030572/s1, Figure S1: Summary of articles included in the review.
