# Supplementary material for: Pharmacokinetics and Pharmacodynamics of Intranasal Solid Lipid Nanoparticles and Nanostructured Lipid Carriers for Nose-to-Brain Delivery
Source: Pharmaceutics. 2022 Mar 5;14(3):572. doi: 10.3390/pharmaceutics14030572 (PMC8948700; doi:10.3390/pharmaceutics14030572)
Supplement: Supplementary file 1 [file pharmaceutics-14-00572-s001.zip › pharmaceutics-1612558-supplementary.pdf]

pharmaceutics

Supplementary Materials: Pharmacokinetics and Pharmacodynamics of Intranasal Solid Lipid Nanoparticles and Nanostructured Lipid Carriers for Nose-to-brain Delivery

Thi-Thao-Linh Nguyen and Han-Joo Maeng

Supplementary information

1. Supplementary method

Search method

We searched three electronic databases, including (i) MEDLINE (PubMed), (ii) Scopus, and (iii) Web of Science, for original studies published up to January 20, 2022. The search terms used were (i) (SLN OR NLC OR "solid lipid nanoparticle" OR "nanostructured lipid carrier" OR "nanostructured-lipid carrier") AND (nose-to-brain OR "nose to brain" OR nasal OR intranasal) for PubMed, (ii) TITLE-ABS-KEY ((SLN OR NLS OR "solid lipid nanoparticle" OR "nanostructured lipid carrier" OR "nanostructured-lipid carrier") AND (nose-to-brain OR "nose to brain" OR nasal OR intranasal)) for Scopus, and (iii) (ALL=(SLN$) OR ALL=(NLC$) OR ALL=(solid lipid nanoparticle$) OR ALL=(nanostructured lipid carrier$) OR ALL=(nanostructured-lipid carrier$)) AND (ALL=(nose-to-brain) OR ALL=(nose to brain) OR ALL=(nasal) OR ALL=(intranasal)) for the Web of Science database. Articles were screened for title, abstract, and content. Review articles, articles without SLNs or NLCs, articles not involving nose-to-brain delivery, articles without an *in vivo* study, articles not written in English, and articles without full-text were excluded. Data were screened by one author and then reviewed by another author.

2. Supplementary figure

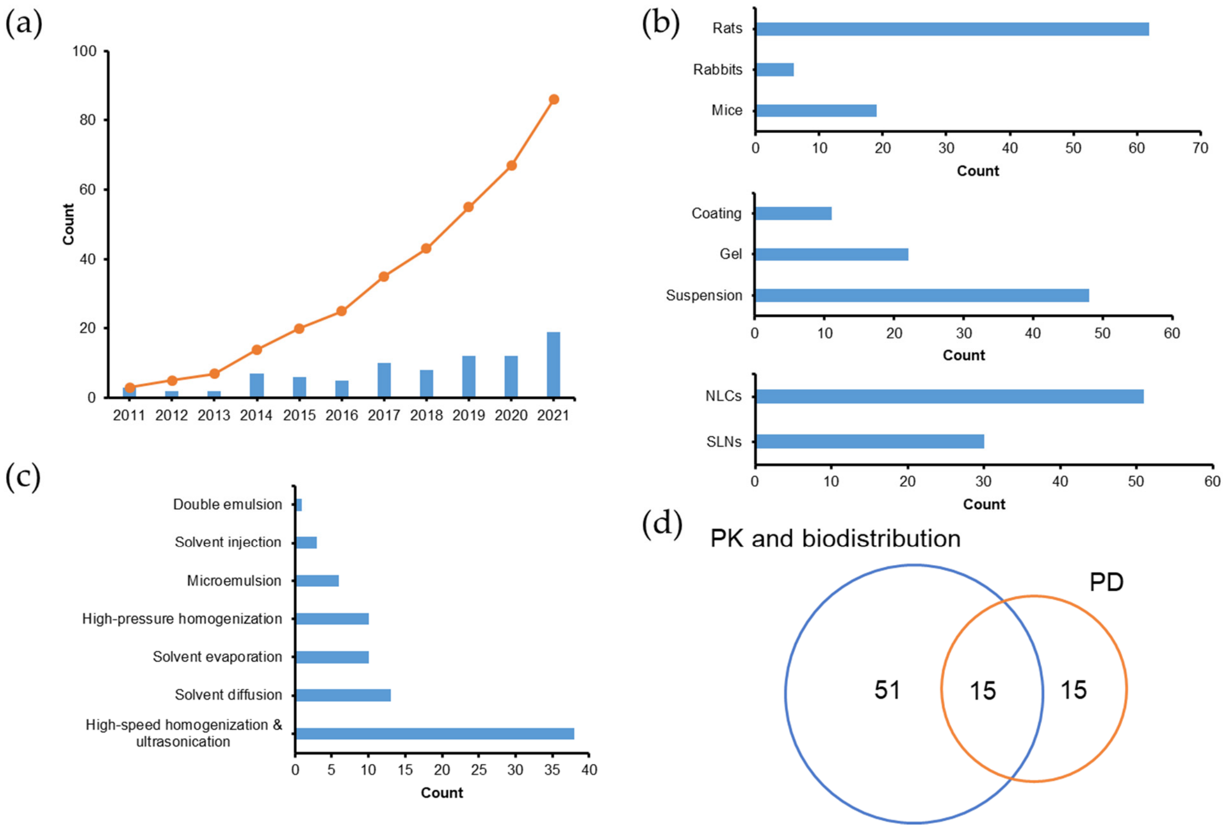

Figure S1. Summary of articles included in the review. (a) Number of articles published by year (blue columns) and cumulative number of articles published (closed circles). (b) Types of animals and formulations. (c) Preparation methods for producing SLNs and NLCs. (d) Types of *in vivo* studies: pharmacodynamic (PD), pharmacokinetic (PK) and biodistribution.
